# Targeting Tryptophan Catabolism in Cancer Immunotherapy Era: Challenges and Perspectives

**DOI:** 10.3389/fimmu.2022.807271

**Published:** 2022-01-31

**Authors:** Florent Peyraud, Jean-Philippe Guegan, Dominique Bodet, Sophie Cousin, Alban Bessede, Antoine Italiano

**Affiliations:** ^1^ Department of Medical Oncology, Institut Bergonié, Bordeaux, France; ^2^ Early Phase Trials and Sarcoma Unit, Institut Bergonié, Bordeaux, France; ^3^ University of Bordeaux, Bordeaux, France; ^4^ Explicyte, Bordeaux, France

**Keywords:** indoleamine 2,3-dioxygenase, cancer, tryptophan metabolism, kynurenine, immunotherapy

## Abstract

Metabolism of tryptophan (Trp), an essential amino acid, represent a major metabolic pathway that both promotes tumor cell intrinsic malignant properties as well as restricts antitumour immunity, thus emerging as a drug development target for cancer immunotherapy. Three cytosolic enzymes, namely indoleamine 2,3-dioxygenase 1 (IDO1), IDO2 and tryptophan 2,3-dioxygenase (TDO2), catalyzes the first-rate limiting step of the degradation of Trp to kynurenine (Kyn) and modulates immunity toward immunosuppression mainly through the aryl hydrocarbon receptor (AhR) activation in numerous types of cancer. By restoring antitumor immune responses and synergizing with other immunotherapies, the encouraging preclinical data of IDO1 inhibitors has dramatically failed to translate into clinical success when combined with immune checkpoints inhibitors, reigniting the debate of combinatorial approach. In this review, we i) provide comprehensive evidences on immunomodulatory role of the Trp catabolism metabolites that highlight this pathway as relevant target in immuno-oncology, ii)ii) discuss underwhelming results from clinical trials investigating efficacy of IDO1 inhibitors and underlying mechanisms that might have contributed to this failure, and finally, iii) discuss the current state-of-art surrounding alternative approaches of innovative antitumor immunotherapies that target molecules of Trp catabolism as well as challenges and perspectives in the era of immunotherapy.

## Introduction

Over the last decade, tumor immune escape has been envisioned as a new paradigm that contributes to tumor growth and evasion, thus appearing as an attractive therapeutic strategy (1). In this regard, new advances in immunotherapies with immune checkpoint inhibitors (ICIs), including drugs targeting the programmed death receptor 1 (PD-1) or its ligand 1 (PD-L1) and cytotoxic T lymphocyte associated protein 4 (CTLA-4), has revolutionized the oncology field and afforded patients with various types of cancer the potential for long-term survival (2). Historically, anticancer therapies (such as chemotherapy, radiotherapy, and targeted therapy) therapeutically act by direct elimination of proliferating cancer cells. Traditionally, their efficacy has been hindered by limiting toxicity and development of resistance. In contrast, the aim of anticancer immunotherapies is to exploit the immune system to eradicate tumor cells and control disease progression. The broad clinical use of ICIs highlights the success in struggling cancer by enhancing T cell-mediated adaptative antitumor immune response.

Tumor cells acquire the ability to hinder effector T cell expansion and function through several mechanisms including the upregulation PD-L1, a major immune checkpoint molecule. Similarly, PD-1 and CTLA-4 are frequently expressed by various immune cells in the tumor microenvironment (TME) and negatively regulate the functions of effector T cells. Thereby, ICIs prevent engagement of PD-1 and CTLA-4 with their corresponding ligands, enabling the immune system to mount potent antitumor immune response by reinvigorating T cells against cancer cells. Consequently, ICIs in clinical care has delivered meaningful clinical benefit across multiple solid tumors. Despite the widening approval of ICIs against an ever growing list of malignancies, the majority of patients do not derive benefit or develop early resistance (3). Patients identified as likely to respond from ICIs include those with tumors harboring a T-cell inflamed phenotype in the TME, suggesting immunosuppressive mechanisms that contribute to ICI failure (4–6). Therefore, novel strategies capable of converting immunosuppressive TME to inflamed tumors may augur a paradigm shift in cancer immunotherapy.

Specific clinical focus has increasingly centered on the metabolic mechanisms of tumor-associated immunosuppression exerted by the tryptophan-kynurenine-aryl hydrocarbon receptor (Trp-Kyn-AhR) pathway in the TME (7). The Trp catabolism is physiologically regulated by indoleamine 2,3-dioxygenase 1 (IDO1), IDO2, and tryptophan 2,3-dioxygenase (TDO2) producing Trp catabolites that can serve as AhR agonists. Pivotal fundamental studies has demonstrated that Kyn is a mainstay signaling molecule that can convey the immunosuppressive effects of IDO1 and TDO2 (8). In preclinical models, enhanced activity of the kynurenine pathway (KP) has been correlated to failing of antitumor immunity and tumor progression which more likely relies on AhR activation (9–13). Recently, the characterization of AhR has revealed several mechanisms by which it engenders a tolerogenic immune milieu (14). Therefore, Trp catabolism pathway is considered as a critical step in tumor cell evasion of both innate and adaptative immune systems (15, 16). Besides, IDO1 has demonstrated a role in mechanisms of resistance to ICIs (17), making the combination of an ICI and IDO1 inhibitor as attractive strategy. To date, the multiple IDO1 inhibitors tested alone or combined with ICI in clinical trials have provided disillusioning results. However, deeper biological and pharmacological insights in Trp catabolism have brought renewed hopes in the anticancer immunotherapy era.

In this review, we first summarize the immunomodulatory role of the Trp-Kyn-AhR pathway. We then provide updated informations on recent advances in the development of innovative anticancer immunotherapies targeting the Trp catabolism and finally discuss upon the current challenges and future perspectives in the field of immuno-oncology.

## Immunomodulatory Role of the Trp Catabolism Pathway

**Figure 1:**
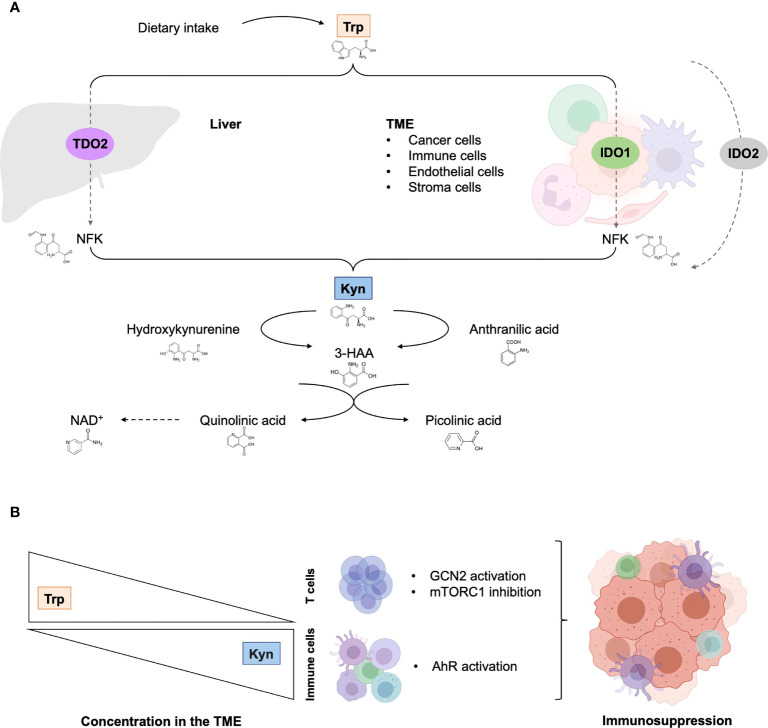
The tryptophan catabolic pathway in cancer. **(A)** Provided by dietary intake, the essential amino acid Trp is catabolized into Kyn through three rate-limiting enzymes: TDO in the liver and IDO1/IDO2 in peripheral tissues. In tumor, IDO1 transforms Trp to Kyn by cleaving the 2,3-double bond of the indole ring, producing N-formyl kynurenine (NFK) that becomes rapidly and spontaneously converted into Kyn. The latter catabolite is further converted into downstream active intermediates, including hydroxykynurenine, anthranilic acid, 3-HAA, quinolinic acid and picolinic acid. The end-products of the pathway are NAD^+^ and others molecules that fuel cellular metabolism. **(B)** Depletion of Trp in T cells suppresses activity in the mTORC1 signaling pathway and activates GCN2, inducing T cell dysfunction and leading to tumor-associated immunosuppression. Increase Kyn level in the TME leads to AhR in multiple tumor-associated immune cells, promoting immunosuppression functions. 3-HAA, 3-hydroxyanthranililic acid; AhR, aryl hydrocarbon receptor; GCN2, general control over nonderepressible 2; IDO1, indoleamine 2,3-dioxygenase (IDO1); Kyn, kynurenine; NAD^+^, nicotinamide adenine dinucleotide; mTORC1, mammalian target of rapamycin complex 1; NFK, N-formyl kynurenine; TDO, tryptophan 2,3-dioxygenase; TME, tumor microenvironment; Trp, tryptophan.

Trp is an essential amino acid that must be taken from the diet to support physiological processes, including cell growth and maintenance (18). It is generally exploited in three main metabolic routes: production of proteins, incorporation into serotonin anabolism, and transformation into kynurenine (Kyn). Three enzymes catalyze the rate-limiting step of the KP that transforms Trp into a series of biologically active metabolites: IDO1, IDO2 and TDO2 (**Figure 1**). Contrary to the uncertain role and regulation of IDO2, IDO1 and TDO2 are well-characterized intracellular heme-containing metalloproteins that are responsible for the degradation of the majority of Trp intake (19).

IDO1 is an inducible enzyme that is expressed widely in many tissues, including endothelial cells, as well as immune cells that are components of the TME, such as dendritic cells (DCs), macrophages, and myeloid-derived suppressor cells (MDSCs). In addition to its recognized catalytic function depriving Trp levels, IDO1 also acts as an intracellular transducer that initiates a series of downstream molecular events, including upregulation of its own expression, activation of the noncanonical nuclear factor kB (NF-kB) pathway and production of the immunoregulatory cytokine transforming growth factor-ß (TGFß) (20, 21). IDO1 recognizes a wide range of indole-containing molecules and is being upregulated by the interplay of inflammatory cytokines, including the pivotal immune regulatory Th1 cytokine interferon γ (IFN-γ) and interleukin-6 (IL-6) (22–24).

By contrast, TDO2 is mainly expressed by hepatocytes and presumably plays a key role in homeostasis of Trp levels as specific enzyme for metabolizing Trp to Kyn. TDO2 is regulated by Trp, cholesterol and prostaglandin E2 (PGE_2_) (25). As a part of concerted mechanisms that occur in the TME, a multitude of cancer cells constitutively express or upregulate IDO1, TDO2, or both, and coerce stromal and tumor-infiltrating immune cells to express IDO1, supporting evasion of immunosurveillance (11, 26–30). To note, additional Trp-derived biogenic amine have been suggested to be crucial component of immune tolerance (31).

**Figure 2:**
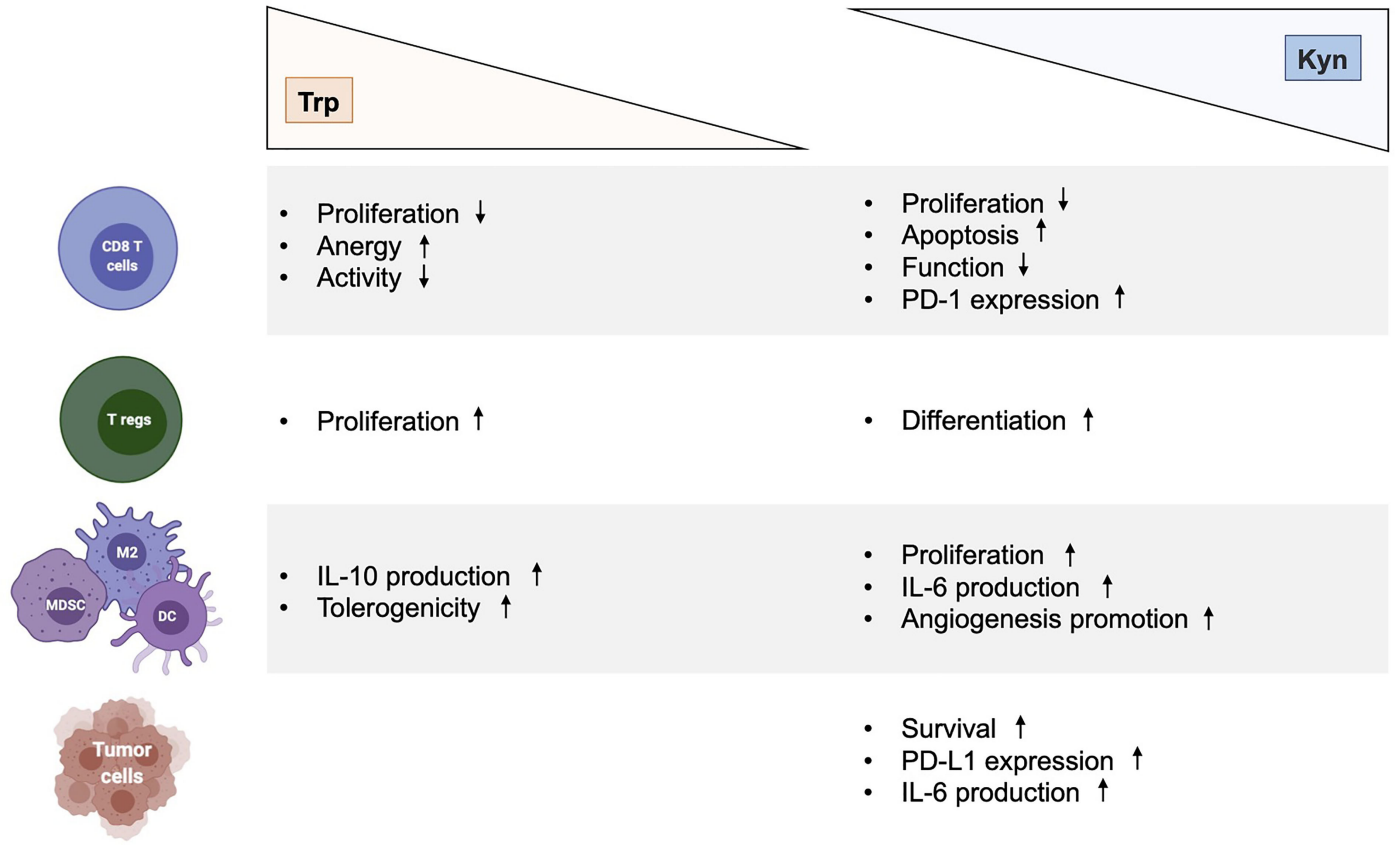
Immunological effects of kynurenine and tryptophan metabolites in cancer. Effects of Trp depletion and Kyn augmentation on CD8 T cells, regulatory T cells, myeloid cells (MDSC, M2, DC) and tumor cells. DC, dendritic cell; Kyn, kynurenine; IL-6, interleukin 6; MDSC, myeloid-derived suppressor cell; M2, type 2 macrophage; Trp, tryptophan.

The multifaceted role and function of Trp catabolic pathway in tumor-associated immunosuppression has been suggested through various mechanisms (**Figure 2**). First, Trp depletion induced by IDO1 in the TME suppress the mammalian target of rapamycin complex (mTORC) pathway and activates the stress response kinase general control over nonderepressible 2 (GCN2) in tumor-infiltrating T cells, leading to their anergy and apoptosis (32–34). In response to low levels of Trp, the serine/threonine kinase GCN2 binds uncharged tRNA and its activated kinase domain phosphorylates eukaryotic initiation factor 2α kinase (eIF2α), resulting in decreased protein production in tumor-infiltrating T cells (35). Moreover, IDO1-mediated GCN2 activation inhibits fatty acid production in naïve T cells, which is necessary for T cell proliferation and activity (36). In addition, activation of GCN2 and inactivation of mTORC contributes to the differentiation, activation and maintenance of the suppressive state of regulatory CD4 T (Tregs) cells that further contribute to tumor-associated immunosuppression (35, 37). However, recent studies have questioned the hypothesis by which inhibition of antitumor immune responses by Trp catabolism is due to local Trp starvation and consequently GCN2-mediated T cell anergy. In preclinical mouse melanoma models, GCN2-deficient T cells demonstrated similar antitumor effect to wild-type T cells against B16 melanoma cell line, highlighting that immune regulation by GCN2 is presumably context-dependent (38). Moreover, GCN2 is a common sensor of amino acid starvation that is not restricted to Trp (39). Thus, additional studies are warranted to elucidate the specific role and function of GCN2 in Trp depletion-associated immunosuppression.

A second postulation has emerged when the Trp catabolite Kyn and its downstream metabolites have emerged as potent endogenous ligand of the AhR (11, 14, 40–42). The ligand-activated transcription factor AhR is an intracellular sensor that integrates signals from various origin (environment, diet, microbiome and metabolic) to control complex transcriptional programs through a ligand-specific, cell-type-specific and context-specific mechanisms. Upon agonist binding, AhR translocates from cytosol to the nucleus and interacts with the AhR nuclear translocator (ARNT). This AhR-ARNT complex is recruited to target genes harboring AhR-responsive elements [known as xenobiotic response elements (XREs)] in their regulatory regions, leading to gene transcription (43). The AhR-ARNT complex is negatively regulated by the AhR repressor (AhRR), which competes with AhR for its interaction with ARNT, thus limiting AhR-driven gene expression (44). Additionally, AhR also controls the expression of genes through non-XRE DNA-responsive elements by interacting with other transcription factors, such as estrogen receptor (ESR) or Krüppel-like factor 6 (KLF6) (43). Moreover, AhR has been described to possess an E3 ubiquitin ligase function that targets various transcription factor, promoting the proteasomal degradation of target proteins. Of note, this dual functions of AhR is modulated by ARNT levels, implying a context-specific activity (45). In addition, AhR has been reported to regulate the activation of other transcription factor, such as NF-kB and signal transducer and activator of transcription (STAT), through direct and indirect pathways (46, 47). Furthermore, AhR has been shown to modulate epigenetic mechanisms, suggesting wider effects on genome (43). Finally, ligand-activated AhR triggers protein phosphorylation driven by SRC upon its release from AhR chaperone complex, including IDO1 (13). Collectively, these observations extend the set of signaling pathways controlled by AhR that modulates biological processes, including the development of neoplastic conditions. Dozens of endogenous AhR agonists have been identified and mainly originate from the diet, enzymatic activities in the commensal flora and host metabolism (**Table 1**). These different AhR ligands confer specificity for pathways and promoters, and hence complicate relevant translational research. While over 90% of dietary Trp is metabolized along the Kyn pathway, a recent study reveals that Kyn derivatives, identified as trace-extended aromatic condensation products (TEACOPS), activated AhR with higher affinity than their precursors (48). However, multiple interactions exist between the gut microbiome and host metabolism that provide multiple AhR agonists, thus requiring deeper understandings to decipher specific programmes controlled by AhR.

**Table 1:** Endogenous AhR ligands, pathways and immune response.

Ligand	Origin	Activity	Consequence
***Tryptophan-derived metabolites***			
Kyn	IDO1, IDO2 and TDO	TAM	Upregulation of IL-6
		Teff	Upregulation of PD-1
		Treg	Differenciation
			Production of IL-10
		Tumor cells	Upregulation of IL-6
		NK	Production of IL-10 and IFN-g
		DC	Expression of CD39 and IDO1
		ILC	–
		Th17	Production of IL-17 and IL-22
		Th22	–
ITE	Tryptophan and cysteine	Naive T cell	Differenciation into Treg
Indole-3-aldehyde, indole-3-acetid acid, indole-3-acetaldehyde, tryptamine, 3-methylindole	Microbiota metabolic product	–	–
FICZ	Ultraviolet B	Naive T cell	Generation and differenciation into Th17
		NK	Production of IFN-g
			Antitumor effect
***Dietary-derived metabolites***			
Indole-3-acetonitrile, indole-3-carbinole, 3,3'-diindolylmethane, indolo (3,4)bicarbazole	Cruciferous vegetables	–	–
***Other metabolites***			
Bilirubin, biliverdin	Haem metabolism	–	–
PGE2, PGG2, leukotriene B4	Arachidonic acid derivatives	–	–

DC, dentritic cell; FICZ, 6-formylindolo[3,2-b]carbazole; IDO, indoleamine 2,3-dioxygenase; IL, interleukine; ILC, innate lymphoid cell; INF-g, interferon gamma; IRF, interferon regulatory factor; ITE, 2-(1’H-indole-3’-carbonyl)-thiazole-4-carboxylic acid methyl ester; Kyn , kynurenine; NK, natural killer cell; PD-1, programmed cell death protein 1; PGE2, prostaglandin E2; PGG2, prostaglandin G2; TAM, tumor-associated macrophage; TDO, tryptophan 2,3-dioxygenase; Teff, effector T cell; Th, helper T cell; TiPARP, 2,3,7,8-tetrachlorodibenzodioxin (TCCD)-inducible poly(ADP-ribose) polymerase; Treg, regulatory T cell.

Numerous researches have revealed a central and complex function of AhR in inducing tolerogenic immunity (14, 43). Indeed, AhR regulates the functions of multiple cells of innate as well as adaptative immune system within a specific context, including natural killer (NK) cells, DCs, macrophages, innate lymphoid cells (ILCs), Tregs, type 17 helper (Th17) cells and Th22 cells. Moreover, AhR activation by Kyn has revealed to promote upregulation of PD-1 expression in CD8 effector T cells and to favor the differentiation of Tregs cells, thereby contributing to the generation of an immunosuppressive milieu (42, 49–51). Kyn-mediated AhR activation also upregulates the expression of genes encoding IL-6 in cancer cells and macrophages, a proinflammatory cytokine (24, 47). The immunosuppressive PGE2 and the IL-6 promote the activity of both TDO2 and IDO1, respectively, which enhance signaling by downstream Kyn and AhR. In the TME, AhR suppresses the immunogenicity of DCs and promote the production of IL-10 by NKs, a potent anti-inflammatory cytokine (41, 52, 53). In tumor-associated macrophages (TAMs), activation of AhR by Kyn contributes to a immunosuppressive phenotype by modulating the expression of tolerogenic molecules such as the ectonucleotidase CD39 (54). The action of AhR in NK cells also promotes the production of IFN-γ, which in turn can upregulate IDO1 expression (52). The interaction of AhR with c-Maf, a transcription factor, furthers the proliferation of Tregs, another major source of IL-10 (55). IL-10 contributes to the development and maintenance of Tregs, which impedes the antitumor function of cytotoxic CD8 T cells (CTLs) (56). IL-10 also promotes the differentiation of tolerogenic DCs (tDCs), tumor-associated macrophages (TAMs), and MDSCs, which invade the TME to promote neovascularization and tumor immune escape (57, 58). Furthermore, the DCs-mediated production of type I IFN potently induces IDO1, which serves as an autonomous feedback control of type I IFN, thus antagonizing its antitumor activities (59). Similarly, type I IFN responses are also negatively regulated by AhR activation, which upregulates the expression of the 2,3,7,8-tetrachlorodibenzodioxin (TCCD)-inducible poly(ADP-ribose) polymerase (TiPARP), leading to inactivation of interferon regulatory factors (IRFs). As a result, the interferon-β (IFN- β) response that is necessary for antitumor immune response, is impaired (60). In addition to the formation of Kyn-related ligands, AhR is also modulated by various endogenous AhR agonists, including 6-formylindolo[3,2-b]carbazole (FICZ) and 2-(1’H-indole-3’-carbonyl)-thiazole-4-carboxylic acid methyl ester (ITE). *In vitro*, treatment of naïve T lymphocyte with FICZ, a high-affinity AhR ligand, favored its differentiation into inflammatory Th17 cells, thus increasing the severity of experimental autoimmune encephalomyelitis in mice (61). In contrast, using the same experimental design, treatment with TCDD favored the induction of Tregs (60). Similarly, *in vivo* ITE-mediated activation of AhR promotes elevation of circulating and intrasplenic Treg of mice, thus highlighting a ligand-dependent properties of AhR on CD4+ T cell fate (60, 62). Altogether, the activation of AhR by Trp-derivative ligands mitigates the innate and adaptative immune responses to lend further support to tumor growth.

Besides the immunomodulatory effects of ligand-activated AhR that jeopardize the tumoricidal immunity, a contrarian view suggests that AhR activation may also drive an antitumor immune response (63). *In vitro* and mouse model of AhR-expressing NK cells demonstrated enhanced IFN-γ production and cytolytic activity when treated with FICZ. In addition, the activation of AhR by FICZ in NK cells impeded the growth of tumor cells in a wild-type immunocompetent mice model of lymphoma and T-cell deficient mice model of melanoma (64). Similarly, treatment of human NK cells with FICZ combined with IL-2 increased degranulation and cytotoxicity (65). In myeloid cells, ligand-activated AhR turned the polarization of monocyte differentiation toward monocyte-derived DCs over monocyte-derived macrophages, thus promoting the production of IL-23 and the development of Th17 cells (66). These opposite networks suggest that immune responses induced by AhR depends on the context and the ligand, thus requiring further investigations. Taken together, these preclinical results unveil complex signaling pathways between the Trp catabolic enzymes IDO1and TDO2, the Kyn and the ligand-operated transcription factor AhR, which is dysregulated by cancer cells to highjack antitumor immunity and develop resistance to anticancer therapies, particularly in the immunotherapy era.

It is of note that Trp-Kyn-AhR pathway correlates with additional immune checkpoints, such as PD-L1 and CTLA-4, supporting the concept of their combined targeting for synergistic antitumor effect (67). In addition, both pathways are induced by IFN-γ in the TME, suggesting a strong association. In response to nonself antigens, such as tumor-associated antigen, the type II interferon – IFN-γ – is secreted by CTLs, which directly promote their antitumor effect. Conversely, IFN-γ also induces the expression of IDO1 and PD-L1, thus attenuating the cytotoxicity of CTLs (68). Previous findings further demonstrated an interplay between IDO1 with both CTLA-4 and PD1/PD-L1 through complex pathways. In mouse model, Tregs induced the expression of IDO1 and Trp catabolism in DCs through a CTLA-4-dependent mechanism (69). Moreover, tolerogenic IDO1-positive DCs are induced by PD-1-expressing mast cells that further stimulates Tregs (70). In addition, PD-1 signaling acted to stabilize IDO1-activated Treg in tumors and prevented them from transforming into inflammatory cells (71). In addition, the expression of IDO1 in the tumor microenvironnement of ovarian cancer promoted PD-1 expression in T cells *via* AhR activation (72). Moreover, the interaction of Kyn with AhR has been shown to stimulate PD-1 expression by CD8 T cells in the TME, which could potentially be reinvigorated by anti-PD-1/PD-L1 drugs (49). Similarly, cancer cells simultaneously activate several immunosuppressive pathways. Indeed, the combination of IDO1 inhibitor with CTLA-4 and PD-1/L1 antibodies demonstrated synergistic antitumor activity in glioma and melanoma mice models (17, 73, 74). In mouse model of melanoma, cancer cells develops resistance to anti-CTLA4 therapy by the upregulation of IDO1, and CTLA-4 antibody strongly synergized with IDO1 inhibitors to mediate elimination of IDO1-positive and negative immunogenic tumors in a T cell dependent manner, underlining the importance of the inhibitory role of both tumor- and host-associated IDO1 (73). Moreover, tumor-associated *IDO1* gene expression strongly correlated with the expression of PD-1, and with increasing level of IFN-γ-responsive gene expression from non-T cell-inflamed to highly T cell-inflamed tumors across multiple human solid tumors from The Cancer Genome Atlas (TCGA) (75). To note, expression of *IDO2*, *TDO*, *KYNU*, *AHR*, and *GCN2* did not correlate with *PD-1* expression or demonstrate IFN-γ responsiveness on a transcriptional level (75). Mounting evidence illuminates IDO1 as a pivotal player that bridges inflammation, vascularization and immune escape, and drives the failure of ICI (73, 76). In clinical care, several IDO1 inhibitors have predominantly been evaluated in combination with other therapies, such as ICI, but providing disappointing results. Multiple approaches are ongoing to target the IDO1/TDO2-Kyn-AhR signaling circuitry, notably in combination with immunotherapy.

## Clinical Development of IDO1 Inhibitors and Trp-Kyn-AhR Pathway Modulators

Within the TME, IDO1 is broadly expressed in multiple human cancers in tumor, vascular, stromal and immune cells. Moreover, IDO1 expression tends to be associated with poorer prognosis (29, 77, 78). Given the critical biological importance of IDO1 in antitumor immunity, IDO1 and its surrounding pathways have been considered as an attractive target. Besides amount number of selective IDO1 inhibitors reported, multiple biochemical modalities have been developed to inhibit the Trp-Kyn-AhR pathway, aiming at regulate mediators of antitumor immunity. Thus, combination with ICI has held great promises, aiming to improve efficacy or to overcome resistance to ICI. To date, IDO1 inhibitors have predominantly been tested in clinical trials alone as well as combined with other treatment modalities, such as ICIs (**Table 2**). Additional pharmacological candidates have entered in clinical development and focus on targeting downstream of IDO1/TDO2. We will outline three different strategies that tackle the IDO1/TDO2-Kyn-AhR signaling circuitry in cancer treatment: (i) pharmacological inhibition of IDO1/TDO2 by IDO1 inhibitors, (ii), systemic depletion of Kyn by engineered kynureninase, and (iii) blockade of AhR activation by synthetic AhR modulators (**Table 3**).

**Table 2:** Clinical trials of IDO1 inhibitors.

Mechanism	Drug	Target	Design	Immunotherapy	Phase	Cancer type	Patients	Intervention	Results	Study identifier
**IDO1 inhibitors**	Epacadostat (INCB024360)	Potent and selective competitive IDO1 inhibitor	Monotherapy	–	I	Advanced solid malignancies	52	Dose escalation (50mg QD or 50, 100, 300, 400, 500, 600, 700mg BID)	- Normalization of Kyn levels at doses ≥ 100mg - No ORR, 7 SD (13.5%)	NCT01195311
					II	Myelodysplastic syndromes	15	600mg BID for 16 wk	- No hematological improvement, 12 SD (80%)	NCT01822691
					II	Advanced epithelial ovarian, primary peritoneal, or fallopian tube cancer	22	600mg BID (vs. Tamoxifen 20mg BID)	- No efficacy difference (mPFS 3.75 vs. 5.56 months, P = 0.54)	NCT01685255
			Combination with immunotherapy	Pembrolizumab	I/II	Advanced solid malignancies (DLBCL, NSCLC, TNBC, HNSCC, UC, RCC, ovarian cancer, endometrial adenocarcinoma, MSI-high CRC, gastric cancer, HCC)	244	100mg BID (vs. Pembrolizumab 200mg Q3W)	- Acceptable safety profile - ORR 35%, DCR 60% in NSCLC cohort - ORR 34%, DCR 62% in HNSCC cohort - ORR 8%, DCR 35% in ovarian cohort - ORR 47%, DCR 58% in RCC cohort - ORR 10%, DCR 36% in TNBC cohort - ORR 35%, DCR 57% in UC cohort	ECHO-202/KEYNOTE-037 (NCT02178722)
					III	Unresectable or metastatic melanoma	706	100mg BID + Pembrolizumab 200mg Q3W (vs. Placebo + Pembrolizumab 200mg Q3W)	- No PFS difference (mPFS 4.7 vs. 4.9 months, HR 1.00, P = 0.52) - No OS difference (mOS NR, HR 1.13, P = 0.81)	ECHO-301/KEYNOTE-252 (NCT02752074)
					II	Advanced or metastatic HNSCC previously treated with a PD-1/PD-L1 inhibitor	–	300mg BID + Pembrolizumab 200mg Q3W	Study withdrawn	NCT03238638
					I	Advanced solid malignancies	6	25 or 100mg BID + Pembrolizumab 200mg Q3W	- Acceptable safety profile - 13.3% of grade 3/4 TRAEs	KEYNOTE-434 (NCT02862457)
				Nivolumab	I/II	Advanced solid malignancies (NSCLC, melanoma, ovarian cancer, CRC, HNSCC, BCNHL, glioblastoma)	241	100 or 300mg BID (vs. Nivolumab 240mg Q2W)	- Acceptable safety profile - DCR 70% in HNSCC cohort (300mg) - ORR 75%, DCR 100% in melanoma cohort (100mg) - ORR 18%, DCR 36% in ovarian cohort (300mg) - ORR 4%, DCR 24% in CRC cohort (100mg)	ECHO-204/JEYNOTE-037 (NCT02327078)
				Atezolizumab	I	Previously treated stage IIIB or IV NSCLC and previsouly treated stage IV UC	29	25mg BID + Atezolizumab 1200mg Q3W	Study terminated (halted prematurely)	ECHO-110 (NCT02298153)
				Ipilimumab	I/II	Unresectable or metastatic melanoma	50	Dose escalation + Ipilimumab 3mg/kg Q3W	- ORR 26% - DLT with ≥ 100 mg BID - IDO1 inhibition at ≥ 25 mg BID	NCT01604889
				MELITAC 12.1 multipeptide vaccine	II	Stage IIIB-IV melanoma	11	300mg BID + MELITAC 12.1 D21, 28, 35, 56, 77, 98	- PR 9%, SD 27% - 91% of Kyn/Trp ratios normalization - Enhanced CD8 T cell infiltrtion	NCT01961115
				CRS-207	I/II	Platinum resistant ovarian, fallopian or peritoneal cancer	35	100mg BID + CRS-207 Q3W	Study terminated (halted prematurely)	SEASCAPE (NCT02575807)
	Navoximod (NLG-919 or GDC-0919)	Non competitive IDO1 inhibitor	Monotherapy	–	IA	Advanced solid malignancies	22	Dose escalation (50 to 1000mg BID)	- Acceptable safety profile - Decreased plasma Kyn	NCT02048709
			Combination with immunotherapy	Atezolizumab	I	Advanced solid malignancies	157	Dose escalation (50 to 1000mg) + Atezolizumab 1200mg Q3W	- PR 9%, ORR 10%, SD 24% - Decreased plasma Kyn with increasing doses	NCT02471846
	Linrodostat (BMS-986205 or F001287)	Potent and selective competitive IDO1 inhibitor	Combination with immunotherapy	Nivolumab or Nivolumab + Ipilimumab	I/IIA	Advanced solid malignancies	907	100mg QD + Nivolumab 240mg Q2W alone or with Ipilimumab	- Acceptable safety profile - ORR 37%, DCR 56% in UC cohort	NCT02658890
	PF-06840003	Potent and selective IDO1 inhibitor	Monotherapy		I	Malignant gliomas	17	Dose escalation (125, 250mg QD or 250, 500 BID)	Study terminated (halted prematurely)	NCT02764151
	LY3381916	Potent and selective IDO1 inhibitor	Combination with immunotherapy	LY3300054 (anti-PD-L1)	I	Advanced solid malignancies	18	240mg QD + LY3300054 700mg Q2W	- Best response: SD	NCT03343613
	Indoximod (1-methyl-D-tryptophan or D-1MT or NLG-8189)	Non competitive IDO1 inhibitor	Combination with immunotherapy	Pembrolizumab or nivolumab or pembrolizumab	II	Advanced melanoma	131	1200mg BID + investigator's choice immunotherapy	- Acceptable safety profile - ORR 51%, DCR 70% - mPFS 12.4 months - PD-L1 positive patients: ORR 70%	NCT02073123

BCNHL, B-cell non-Hodgkin lymphoma; BID, twice a day; CR, complete response; CRC, colorectal cancer; D, day; D-1-MT, 1-methyl-D-tryptophan; DCR, disease control rate (CR+PR+SD); DLBCL, diffuse large B-cell lymphoma; E, epacadostat; HCC, hepatocellular carcinoma; HNSCC, head and neck squamous-cell carcinoma; Kyn, kynurenine; mPFS, median progression-free survival; MSI-high, microsatellite instability; NCT, national clinical trial (https://www.clinicaltrials.gov); NR, not reached; NSCLC, non-small cell lung carcinoma; ORR, objective response rate (CR+PR); PD-L1, programmed cell death ligand 1; PR, partial response; Q2W, every 2 weeks; Q3W, every 3 weeks; QD, once a day; RCC, renal-cell carcinoma; SD, stable disease, TNBC, triple negative breast cancer; TRAE, treatment-related adverse event; Trp, tryptophan; UC, urothelial carcinoma.

**Table 3:** Ongoing clinical trials of Trp metabolism-targeting therapies in combination with immunotherapy.

Mechanism	Drug	Target	Immunotherapy	Phase	Cancer type	Patients	Intervention	Status	Study identifier	NB
										Primary endpoint
**IDO1 inhibitors**	Epacadostat (INCB024360)	Selective competitive IDO1 inhibitor	Pembrolizumab	III	First-line treatment for mRCC	129	Epacadostat + Pembrolizumab (vs. Sunitinib or Pazopanib)	Active, not recruiting	ECHO-302/KEYNOTE-679 (NCT03260894)	Objective Response Rate (ORR) of Pembrolizumab + Epacadostat Versus Standard of Care (SOC) [ Time Frame: Minimum up to 6 months ]
				III	First-line treatment for HNSCC	89	Epacadostat + Pembrolizumab (vs. Pembrolizumab or chemotherapy)	Active, not recruiting	ECHO-304/KEYNOTE-669 (NCT03358472)	
				II	Unresectable or metastatic GEJ and gastric adenocarcinoma	3	Epacadostat + Pembrolizumab	Completed	NCT03196232	Progression-free Survival (PFS) [ Time Frame: 6 months ]
				II	Metastatic pancreatic cancer	44	Epacadostat + Pembrolizumab + CRS-207 +/- Cyclophosphamide + GVAX	Recruiting	NCT03006302	GVAX: GM-CSF gene-transfected tumor cell vaccine
				I/II	Advanced or metastatic solid malignancies	70	Epacadostat + Pembrolizumab + Chemotherapy (among 7 regimens)	Completed	ECHO-207/KEYNOTE-723 (NCT03085914)	Phase 2: Objective Response Rate (ORR) [ Time Frame: Up to Week 18 ]
				II	Thymic carcinoma	45	Epacadostat + Pembrolizumab	Active, not recruiting	NCT02364076	
				II	Sarcoma	30	Epacadostat + Pembrolizumab	Active, not recruiting	NCT03414229	
				II	Unresectable HNSCC	14	Epacadostat + Pembrolizumab + Tavokinogene Telseplasmid with electroporation	Active, not recruiting	NCT03823131	
			Durvalumab	I/II	Advanced solid malignancies (pancreas, melanoma, NSCLC, HNSCC)	176	Epacadostat + Durvalumab	Completed	ECHO-203 (NCT02318277)	Phase 2: Objective Response Rate (ORR) as Determined by Radiographic Disease Assessments Per Modified Response Evaluation Criteria in Solid Tumors (RECIST) v1.1 [ Time Frame: Measured every 8 weeks for duration of study treatment [approximately 12 months] ]
			Retifanlimab (INCMGA00012)	I	Unresectable or metastatic solid tumors	100	Epacadostat + Retifanlimab	Active, not recruiting	POD1UM-102 (NCT03589651)	DEC-205/NY-ESO-1 fusion protein CDX-1401 and poly ICLC (immunostimulant TLR3-agonist)
				II	Advanced or metastatic endometrial cancer	220	Epacadostat + Retifanlimab	Recruiting	POD1UM-204 (NCT04463771)	
				I/II	Locally reccurent or metastatic breast cancer	60	Epacadostat + Retifanlimab + SV-BR-1-GM	Recruiting	NCT03328026	
				II	Recurrent gliomas	55	Retifanlimab + Bevacizumab + Radiotherapy +/- Epacadostat	Recruiting	NCT03532295	
				II	Neoadjuvant urothelial carcinoma	45	Epacadostat + Retifanlimab	Not yet recruiting	OPTIMUS (NCT04586244)	
			CDX-1401 + TLR3 agonist	I/IIb	Ovarian, fallopian tube or primary peritoneal cancer in remission	40	Epacadostat + CDX-1401 + TLR3 agonist (poly ICLC)	Active, not recruiting	NCT02166905	
			Bispecific PD-L1/TGFβ Ab + IL15 agonist + BN-Brachyury vaccine	I/II	Advanced solid malignancies	113	Epacadostat + bispecific PD-L1/TGFβ Ab + IL15 agonist + BN-Brachyury vaccine	Recruiting	QuEST1 (NCT03493945)	
	Linrodostat (BMS-986205 or F001287)	Potent and selective competitive IDO1 inhibitor	Nivolumab	I/II	Advanced or metastatic solid malignancies	12	Linrodostat + Nivolumab	Active, not recruiting	NCT03792750	
				II	Stage II-IV HNSCC		Linrodostat + Nivolumab	Recruiting	NCT03854032	
				II	Recurrent or persistent endometrial carcinoma or endometrial carcinosarcoma	50	Linrodostat + Nivolumab	Recruiting	NCT04106414	
				I/IIa	Advanced or metastatic solid malignancies	516	Linrodostat + Nivolumab +/- Ipilimumab	Recruiting	NCT02658890	
				I/II	Advanced or metastatic solid malignancies	230	Linrodostat + Nivolumab +/- Ipilimumab or Relatlimab	Recruiting	NCT03459222	
				III	Muscle invasive bladder cancer	1200	Chemotherapy +/- Linrodostat +/- Nivolumab	Recruiting	NCT03661320	
				I	Newly diagnosed glioblastoma	30	Linrodostat + Nivolumab + Radiotherapy +/- Temozolomide	Recruiting	NCT04047706	
				II	Advanced mRCC	200	Linrodostat + Nivolumab	Recruiting	FRACTION-RCC (NCT02996110)	
				I	Advanced or metastatic solid malignancies	50	Linrodostat + Nivolumab	Recruiting	ADVISE (NCT03335540)	
				II	BCG-unresponsive non-muscle invasive bladder cancer	69	Linrodostat + Nivolumab +/- BCG therapy	Active, not recruiting	CheckMate 9UT (NCT03519256)	
	KHK2455	Long acting and selective IDO1 inhibitor	Avelumab	I	Locally advanced or metastatic urothelial carcinoma	50	KHK2455 + Avelumab	Recruiting	NCT03915405	
	Navoximod (NLG-919 or GDC-0919)	Non competitive IDO1 inhibitor	–	–	–	–	–	–	–	
	Indoximod (1MT or NLG-8189)	Competitive IDO1 inhibitor (Trp mimetic)	–	–	–	–	–	–	–	
	MK-7162	Selective IDO1 inhibitor	–	–	–	–	–	–	–	
	LY3381916	Selective and potent IDO1 inhibitor	–	–	–	–	–	–	–	
	PF-06840003	Selective IDO1 inhibitor	–	–	–	–	–	–	–	
**Recombinant kynureninases**	–	–	–	–	–	–	–	–	–	
**AhR modulators**	BAY2416964	AhR inhibitor	Pembrolizumab	I	Advanced or metastatic solid malignancies	78	BAY2416964 + Pembrolizumab	Not yet recruiting	NCT04999202	
	IK-175	AhR inhibitor	Nivolumab	I	Advanced or metastatic solid malignancies	93	IK-175 +/- Nivolumab	Recruiting	NCT04200963	
**Others**	IO-102	Single PD-L1/IDO peptide vaccine	Pembrolizumab	I/II	First-line treatment for NSCLC	108	IO-102 + Pembrolizumab +/- Chemotherapy (Carboplatin-Pemetrexed)	Active, not recruiting	NCT03562871	
			Nivolumab	I/II	Naïve and anti-PD-1/PD-L1 refractory metastatic melanoma	50	IO-102 + Nivolumab	Recruiting	NCT03047928	
	SHR9146 (HTI-1090	Dual IDO/TDO inhibitor	Camrelizumab	I	Advanced or metastatic solid malignancies	200	SHR9146 + Camrelizumab	Unknown	NCT03491631	

1MT, 1-methyl-D-tryptophan; Ab, antibody; GEJ, gastroesophageal junction; ICI, immune-checkpoint inhibitors; IDO, indoleamine 2-3-dioxygenase; IL, interleukin; HNSCC, head and neck squamous cell carcinoma; mRCC, metastatic renal cell carcinoma; NSCLC, non-small cell lung cancer; PD-1, programmed cell death protein 1; PD-L1, PD-1 ligand; TLR3, toll-like receptor 3; Trp, tryptophan.

### Clinical Development of IDO1 Inhibitors

Since the first report of indoximod as an IDO1 pathway antagonist, multiple other IDO1 inhibitors have been generated to inhibit the ligation of Kyn with AhR. Except indoximod, selective IDO1 inhibitors enzyme aim to have higher affinity for IDO1 than Trp or to compete the catalytic site of IDO1. Most of these molecules were evaluated in a combinatorial approach with ICI (**Table 2**).

Epacadostat (INCB24360) is an orally available IDO1-selective inhibitor that is still under active investigation in clinic. Competing with Trp for binding to the catalytic domain of IDO1, epacadostat demonstrated weak inhibitory activities against Kyn synthesis in a human enzymatic and blood assays of IDO1, with essentially no activity against TDO2 (79). Inhibition of IDO1 catalytic activity by epacadostat prevented Treg proliferation induced by DCs stimulated with IFN-γ that induced IDO1 expression. In *in vitro* tumor cell lysis assays, T cells stimulation with epacadostat-treated DCs produced significantly more IFN-γ and demonstrated greater potency (80). Moreover, epacadostat promoted the growth of NK cells and effector T cells, reduced conversion of naive T cells to Tregs, and increased the quantity of DCs in co-cultures of human allogeneic lymphocytes with DCs or tumor cells (26). In immunocompetent mice syngeneic models of pancreatic and colorectal cancers, epacadostat was found to inhibit tumor growth. This antitumor efficacy was abolished in immunodeficient mice, supporting an immune-mediated antitumor response (81). The combination of epacadostat with anti-PD-1 antibody significantly improved survival of C57BL/6 mice implanted intracranially with murine GL261 glioblastoma, suggesting a potential synergistic effect of the combinatorial treatment (82). In the first-in-human phase I clinical trial conducted in patient with cancer receiving epacadostat, Kyn concentration in plasma indicated 80-90% inhibition of IDO activity at dose levels at or above 100mg twice daily. Although plasma Kyn was decreased at all dose levels, no changes in plasma proteins related to immunity or inflammation could be detected. To note, no objective response was observed in advanced cancer patients when dosed as monotherapy, potentially suggesting insufficient dosing (83). This lack of activity was evidenced in two phase II studies in patients with myelodysplastic syndrome or advanced ovarian cancer, leading to the development of IDO1 inhibitors combined with conventional anticancer medications (84, 85). Owing to the strong rational to combine an IDO1 inhibitor with ICI, ipilimumab (anti-CTLA4 antibody), nivolumab (anti-PD-1 antibody), or pembrolizumab (anti-PD-1 antibody) have been evaluated in combination with epacadostat for the treatment of melanoma, non-small cell lung cancer (NSCLC), colorectal cancer (CRC), head and neck squamous cell carcinoma (HNSCC) and renal-cell carcinoma (RCC). Despite the seeming acceptable safety profile and promising preliminary results from phase I/II trials in various tumor types, the combination of epacadostat with ICI have displayed unexpected results in phase III trials. In ECHO-301/KEYNOTE-252, epacadostat 100mg twice daily combined with pembrolizumab did not improve PFS compared to pembrolizumab+placebo (mPFS 4.7 vs. 4.9 months, HR 1.00, P = 0.52) and OS (mOS NR, HR 1.13, P = 0.81) in patients with unresectable or metastatic melanoma (86). Multiple explanations have been raised to understand these discrepancies, including differences between the treated population, inappropriately low dosing of epacadostat, and incomplete suppression of intratumor Kyn. No information regarding the impact of epacadostat on plasma Kyn levels was reported to date.

Navoximod (NLG-919 or GDC-0919) is an orally bioavailable noncompetitive IDO1 inhibitor that potently inhibited IDO1-associated T cell immunosuppression and restored robust T cell responses in *in vitro* assay (87). In preclinical syngeneic mouse melanoma model, combination of navoximod with PD-1/L1 inhibitors generated synergistic antitumor response, where increased CTL/Tregs ratios, circulating IFN-γ levels and activated TAMs and DCs were noted, arguing for clinical development (88, 89). Contrary to the encouraging results from epacadostat in early phase trials, no clear evidence of benefit from coadministration of navoximod (given at 600mg or 1000mg BID) with atezolizumab (anti-PD-L1 antibody) in patients with advanced solid tumors was observed (90). Interestingly, although navoximod decreased plasma Kyn relative to predose levels, no differences in the level of Kyn suppression were observed between responders and nonresponders.

Linrodostat (BMS-986205 or F001287) is considered as the most potent IDO1 selective inhibitors with no activity against TDO2 (91). Linrodostat demonstrated strong activity in *in vitro* experiment of coculture of T cells with IDO1-expressing DCs. In addition to the well-tolerated profile, patients with advanced solid cancers receiving linrodostat plus nivolumab reported substantial serum and intratumoral Kyn reduction, supporting further evaluation in late-stage clinical studies (92). To date, several ongoing trials evaluate Linrodostat with ICI in different type of cancers.

PF-06840003 (EOS200271) is another orally bioavailable and highly selective Trp noncompetitive IDO1 inhibitor that entered first-in-human early phase trial in patients with recurrent malignant glioma (93). One advantage of this candidate compound is its ability to cross the blood-brain barrier that may enable efficient access to brain metastases. Only limited data have been reported but preclinical evidence suggested that PF-06840003 was able to reverse IDO1-mediated T cell anergy *in vitro*, and decreased intratumoral Kyn level in mice (94). The combination of PD-L1 blockade avelumab with PF-06840003 demonstrated inhibition of tumor growth in several syngeneic mouse tumor models but failed to achieve significant efficacy in phase I trial. To date, no study is ongoing with PF-06840003, either alone or in combination with ICI.

In contrast with other IDO1 inhibitors that exert direct inhibitory activity on IDO1 enzyme, indoximod (1-methyl-D-tryptophan, D-1MT, or NLG-8189) is a Trp mimetic with pleiotropic effects on downstream Kyn-AhR pathway signaling. It relieves IDO1-mediated immunosuppression by at least two mechanisms: (i) modulation of AhR-dependent transcriptional activity and (ii) preventing activation of GCN2 and inhibition of mTORC1 by delivering an artificial Trp-sufficiency signal (95–97). Indeed, indoximod enhanced function of tumor-infiltrating effector and helper T cells by inhibiting Trp depletion-associated mTORC1 suppression, thus opposing and bypassing the effects of Trp deprivation that lead to GCN2 activation (97). Indoximod also demonstrated to favor the differentiation of CD4 T cells into Th17 helper T cells, decrease Tregs, and inhibit expression of IDO1 in DCs through an AhR-dependent manner (98). Adding indoximod was shown to safely increase the antitumor activity of anti-PD-1 in melanoma patients (99). In a single-arm phase II trial, the combination of indoximod with anti-PD-1 in advanced melanoma achieved antitumor response (100). Recently, a prodrug structure of indoximod named NLG-802 has been shown to heighten the pharmacological exposure of the drug higher than the current achievable levels, and could potentially widen the therapeutic window in a subset of patients (101).

In addition to the aforementioned IDO1 inhibitors, some other small molecules or vaccines have fared clinical development, including LY3381916, MK-7162, IO102 and KHK2455, alone or combined with ICI. For example, LY3381916 is a highly selective and potent inhibitor of IDO1 that was evaluated alone or combined with a PD-L1 blocker in patients with advanced or metastatic cancer (102). No clear clinical activity was detected, thus warranting further studies. Recently, a first-in-class immune-modulatory vaccine (IO102/IO103) against IDO1 and PD-L1 targeting IDO1 and/or PD-L1-positive immunosuppressive cells and tumor cells was tested in combination with nivolumab in patients with naïve metastatic melanoma. Among the 30 treated patients, the overall response rate was 80% with complete responses in 43% (103). Interestingly, T cell influx of peripherally expanded T cells into tumor sites was observed in responding patients, and general enrichment of IDO- and PD-L1-specific clones after treatment was documented, suggesting a potential of this immunomodulating approach (103).

In the wake of epacadostat failure in late-stage trials, most of IDO1 inhibitors have been scaled back in their clinical development, although randomized studies are still ongoing or planned (**Table 3**). Perhaps of most relevance to explain the failure of IDO1-targeting inhibitors is the question of intratumoral pharmacodynamics.

It is yet unknown whether the limitation of Trp depletion or the suppression of Kyn acts primary to promote immunosuppression. Indeed, even very low levels of canonical AhR ligands are able to promote AhR gene expression, while kynureninase and direct AhR inhibitors display higher potency than IDO-1 selective inhibitors in recent preclinical studies (104, 105).

### Recombinant Kynureninases

Along the KP, Kyn is produced from Trp by the catabolic activity of IDO1 and TDO2, and is catabolized by various enzymes into downstream intracellular metabolites (**Figure 1**). Physiologically, kynureninase is an enzyme that synthetize anthranilic acid from Kyn, and 3-hydroxyanthranilic acid (3-HAA) from 3-hydroxykynurenine. In addition, large neural amino acid transporter are able to capture extracellular Kyn, thus regulating the cellular content of Trp (15).

One emerging strategy relies on the prevention of Kyn engagement with AhR. To this end, the development of recombinant kynureninases aims to enzymatically deplete the extracellular pool of Kyn to limit its intracellular availability, thus hampering the Kyn-AhR interaction that promotes tumor-associated immunosuppression (106–108). Engineered from the bacsterial kynureninase enzyme, recombinant kynureninase more effectively transforms Kyn into immunologically inactive downstream metabolites than does the endogenous kynureninase. Multiple lines of evidence suggest that enzyme-mediated Kyn depletion is a promising cancer immunotherapeutic strategy. In syngeneic mice model of melanoma, depletion of Kyn in both plasma and tumor by Kyn-degrading enzymes kynureninase reduced Kyn levels in IDO1, TDO2 and IDO1/TDO2-expressing cancer cells and augmented effector T cells in tumor, without impact on Trp levels. Beside its direct tumoricidal effect in mice bearing CT26 colon carcinoma, kynureninase treatment induced accumulation of effector CD8 T cells within the tumor nest, as well as higher level of IFN-γ in the TME. Similarly, unique dose of recombinant kynureninase decreased Kyn levels and promoted higher levels of CD8 T cells in mice tumor models, thus modulating the effects of IDO1 and TDO2 within the TME (109). The tumoricidal function of recombinant kynureninase was annihilated in IDO1 knockout mice and in *Rag^-/-^* mice depleted for CD8 T cells, suggesting its addiction on IDO1 and functional immune system, respectively. In melanoma, colon cancer CT26 and breast cancer 4T1 models, combination of engineered kynureninase with ICI exhibited significant tumor growth inhibition and survival benefit. The administration of a pharmacologically optimized kynureninase had substantial therapeutic effects when combined with approved checkpoint inhibitors or with a cancer vaccine for the treatment of 4T1 breast carcinoma, melanoma or CT26 colon carcinoma tumors (109). Moreover, the combination of kynureninase with anti-PD1 was more effective than the latter with epacadostat in colon cancer mice model. Additional studies are warranted to evaluate the safety as well as antitumor activity of engineered kynureninase as single agent or combined with ICI (**Table 3**).

A pharmacologically optimized human kynureninase is currently moving toward clinical development for the treatment of cancers where Trp-Kyn-AhR pathway play a significant immunosuppressive role through Kyn production, and those independently of both IDO1 and TDO2 overexpression.

### Synthetic AhR Modulators

Aggressive malignancies harbor higher expression of AhR, the latter constitutively translocates to the nucleus, suggesting its role in tumor progression (110). It is well-established the activation of AhR in immune cells hinders efficient antitumor immunity *via* stimulation of antigen-presenting DCs, tumor-associated macrophages (TAMs), immunosuppressive Tregs and modulation of effector CD8 and CD4 T-cell functions (111). Beside hampering the modulation of Kyn, inhibition of AhR signaling activation with small-molecule antagonists is an alternative strategy, which seeks to interfere with the immunosuppression functions regardless of the origin of Kyn (112). A major challenge to the development of selective AhR modulators reside in its ligand promiscuity (113). Although numerous diverse metabolites are potent activator of AhR signaling, only a subset compounds are used as AhR antagonists, aiming to understand the immunological roles of AhR (114–116).

Early mouse and human AhR inhibitors have demonstrated to inhibit nuclear localization of AhR and increased secretion of IFN-γ, TNF-α, and IL-2 in *ex vivo* assays. In the CT26 colon carcinoma syngeneic mouse model, AhR blockade with a potent antagonist as monotherapy enhanced T cell function, decreased M2-like macrophages infiltration and drove antitumor immune response *in vivo*, resulting in the inhibition of tumor formation (105). Recently, AhR antagonist exhibited anti-tumor efficacy alone and increased the activity of PD-L1 blockade in various syngeneic mouse tumor models (117). Numerous AhR inhibitors have entered *in vivo* studies. Notably, BAY-218, a selective AhR blocker, increased therapeutic activity of anti-PD-L1 antibody in the CT26 colon carcinoma model (118, 119).

Several companies have disclosed the development of AhR inhibitors in clinic, without biological data in human reported to date. For example, BAY-218 is currently tested in phase I trial for therapy of patients with advanced cancer. However, one caveat resides in the unresolved role of AhR in tumor growth and metastasis. Indeed, activation of AhR by its ligand demonstrated to decrease metastatic process and stemness of breast cancer cells (120–122). Therefore, the antitumor activity of AhR agonist or antagonist remains to be clarified in clinical setting and whether these drugs act directly on cancer cells or by modulating antitumor immunity, thus suggesting synergistic effect when combined with ICI.

## Challenges and Perspectives

Amount of evidence suggest that IDO1- or TDO2-expressing tumor cells can escape immunosurveillance *via* Trp starvation, and AhR is involved in tumor immune evasion. Therefore, there is a strong translational rational for joint therapeutically targeting the Trp-Kyn-AhR axis with immunotherapy. Despite prolonged responses have been observed for epacadostat in combination with pembrolizumab, efficacy was globally lacking from multiple clinical trials evaluating IDO1 inhibition with anti-PD-1/L1-based ICI. These conflicting results were questioned with the recent description of epacadostat and navoximod as AhR agonists, casting doubt on the mechanism of action of these pharmacological compounds (123). While the role of this unexpected activity is uncertain, it may have had a bearing on the immune-activating effect of IDO1 inhibition and have counteracted ICI synergistic action. Therefore, examination of clinical activity of novel IDO1 inhibitors on AhR activity should be made before entering in clinical development. Much more potent and optimized IDO1 inhibitors have entered human clinical trials in combination with immunotherapeutic agents (**Table 3**). In addition, the relevance of non-enzymatic immunomodulatory activity of IDO1 has to be addressed in tumors. Indeed, recent findings suggest that IDO1 in tumor cells and non-tumor cells possess different functions that are non-overlapping, which may have bearing on difference in targetability with first generation of IDO1 inhibitors primarily focused on catalytic activity (124, 125). Therefore, novel agents that both target the enzymatic and signal transduction properties of IDO1 may offer wider therapeutic effect and leverage antitumor immune response.

In parallel, alternative mechanisms are being investigated in order to achieve intratumoral Kyn reduction, aiming to limit the protumoral and immunomodulatory effects of Kyn. Fostering the degradation of circulating Kyn by kynureninase represent a novel approach. In one hand, kynureninase suppress Kyn and prevents the activation of AhR, thus reinstating antitumor immunity. This mechanism might be able to block the protumoral function of IDO1 and TDO. A second new concept is AhR antagonists, which could theoretically inhibit AhR activation by all agonist regardless of their origin. Kynureninases and AhR antagonists represent cutting edge immunometabolic compounds that could safely magnify the benefit of cancer immunotherapy. In comparison to directly targeting IDO1, further investigations are warranted to evaluate the potential synergistic effect between these novel strategies with ICI (126).

Growing evidence supports TDO2 as another Trp catabolic enzyme involved in immune escape and TDO2 inhibition could be a new immunomodulatory approach to strike tumor (111, 127). Nevertheless, TDO2 enzyme conveys different inflammatory characteristics in spite of common role of in Trp metabolism (128). These differences could be explained by enzymological differences as well as variation in locoregional control of Kyn production or effectiveness of Kyn downstream pathways, including AhR activation and KP catabolic enzymes. While no genetic preclinical proof of concept exists to date, there is a pharmacological rational to evaluate the combination of IDO1 and/or TDO inhibitors in combination with TDO2 antagonists as next-generation strategies. In this regard, improvement of pharmacological engineering has led to the development of dual IDO1/TDO2 inhibitors that could indiscriminately depletes intratumoral and systemic Kyn produced by IDO1/TDO2. In preclinical cancer models, the IDO1/TDO inhibitor RG70099 demonstrated significant decrease Kyn levels, while reduced tumor volumes was reported in response to the IDO1/TDO inhibitor EPL-1410 when evaluated as single agent (129, 130). The IDO1/TDO dual inhibitors CB548 and CMG017 elicited a robust antitumor immune response and dampen tumor progression when combined with ICI (131). Owing to encouraging preclinical results, novel compounds from these different classes approach early phase studies or are already ongoing, thus opening new insights into targeting Trp catabolism.

There exist an interplay between tumor cells and the host microbiota that produce AhR agonists from dietary sources. Recently, the gut microbiome has been identified as a major actor in regulating cancer initiation, progression as well as response to ICI (132, 133). The balance between intestinal immune tolerance and gut microbiota is regulated by Trp and its IDO1-catalyzed endogenous metabolites. Recent findings have highlighted that Trp metabolism may modulate microbiota to regulate the host immune system (134). These profound effects of gut microbiota on Trp-associated immune homeostasis might be linked to tumorigenesis and implicated in several cancers as well as resistance to therapies, such as ICI. In this regard, dietary or environmental exposures have to be considered as editable variable in population treated by modulators of Trp-Kyn-AhR axis especially in the light of role of AhR that is able to act as a xenobiotic sensor responsive to gut microbiome-associated signals (14). Therefore, understanding these crosstalks between immune system, tumor progression and gut microbiota is of utmost importance to adapt immunomodulatory drugs, such as ICI and drugs targeting the Trp-Kyn-AhR axis.

Finally, the development of cancer immunotherapy still lacking accurate biomarkers able to predict ICI efficacy. While it is simple to measure Trp and Kyn levels *in vitro*, quantification of IDO1 metabolism is more challenging *in vivo*. Recently, an innovative positron emission tomography imaging of IDO1 has shown to be an accurate method to evaluate the therapeutic activity of combined immunotherapies and tailoring optimal personalized combination strategies (135). In the wake of evaluation of novel inhibitors of Trp-Kyn-AhR axis, assessment of pharmacodynamic endpoints should be considered, such as reduction of circulating or extracellular Kyn within TME.

While prior studies demonstrated a correlation between Kyn/Trp level and response to anti-PD-1, one concern relies on the method used to evaluate the selective IDO1 inhibitor epacadostat, where no intratumoral Kyn biomarker analysis was performed (136, 137). However, it has to be proven whether serum Kyn is a surrogate marker of intratumoral Kyn. In the next generation of clinical trials, it will be warranted to stratify patients based on tumor enzyme expression, Trp catabolite levels in the TME or systemic circulation, and the activation of downstream signaling pathways, aiming to identify those who are more likely to respond.

## Conclusion

Cancer immunotherapy has achieved a great accomplishment with the approval of PD-1/L1 and CTLA-4 inhibitors in the clinical care of a growing list of cancer types. However, only a subset of patients derive benefit, underscoring the need to develop novel strategies to circumvent primary or secondary resistances. Tumor cells use several biological mechanisms to mount an immune permissive milieu and escape from the host antitumor immunity. Activation of the Trp-Kyn-AhR pathway has been recognized as one of such mechanism. The immune suppressive effect of this pathway is considered to be mediated by IDO1/TDO-mediated Trp starvation, Kyn-mediated T cell-associated adaptative immunity dysfunction and subsequent activation AhR. Therefore, combining Trp catabolism-targeting drugs with ICI have brought high expectations in the field of immunotherapy.

To date, the evaluation of IDO1 inhibitors alone or combined with ICI in clinical trials have provided disillusioning results, requiring more comprehensive understandings on the role of Trp-Kyn-AhR pathway in cancer development and immunology. This area remains controversial but highlights the need for effective and physiologically relevant preclinical studies to best identify the conditions in which IDO1 inhibitors and modulation of tryptophan metabolism in the TME may add therapeutic value. Combinatorial and innovative strategies targeting the Trp-Kyn-AhR pathway might allow a special opportunity to extend the therapeutic window of numerous treatments, especially in the era of immunotherapy.

## Author Contributions

FP: conceptualization, methodology, investigation, writing – original draft. JPG: methodology, writing – review & editing. DB: investigation, writing – original draft. SC: investigation, writing – original draft. AB: conceptualization, writing – review & editing, supervision. AI: conceptualization, writing – review & editing, supervision.

## Conflict of Interest

The authors declare that the research was conducted in the absence of any commercial or financial relationships that could be construed as a potential conflict of interest.

## Publisher’s Note

All claims expressed in this article are solely those of the authors and do not necessarily represent those of their affiliated organizations, or those of the publisher, the editors and the reviewers. Any product that may be evaluated in this article, or claim that may be made by its manufacturer, is not guaranteed or endorsed by the publisher.

## References

[1] Schreiber RD Old LJ Smyth MJ. Cancer Immunoediting: Integrating Immunity’s Roles in Cancer Suppression and Promotion. Science (2011) 331:1565–70. doi: 10.1126/science.1203486 21436444

[2] Robert C. A Decade of Immune-Checkpoint Inhibitors in Cancer Therapy. Nat Commun (2020) 11:3801. doi: 10.1038/s41467-020-17670-y 32732879 PMC7393098

[3] Sharma P Hu-Lieskovan S Wargo JA Ribas A. Primary, Adaptive, and Acquired Resistance to Cancer Immunotherapy. Cell (2017) 168:707–23. doi: 10.1016/j.cell.2017.01.017 PMC5391692 28187290

[4] Gajewski TF Meng Y Blank C Brown I Kacha A Kline J. Immune Resistance Orchestrated by the Tumor Microenvironment. Immunol Rev (2006) 213:131–45. doi: 10.1111/j.1600-065X.2006.00442.x 16972901

[5] Spranger S. Mechanisms of Tumor Escape in the Context of the T-Cell-Inflamed and the non-T-Cell-Inflamed Tumor Microenvironment. Int Immunol (2016) 28:383–91. doi: 10.1093/intimm/dxw014 PMC4986232 26989092

[6] Lanitis E Dangaj D Irving M Coukos G. Mechanisms Regulating T-Cell Infiltration and Activity in Solid Tumors. Ann Oncol (2017) 28:xii18–32. doi: 10.1093/annonc/mdx238 29045511

[7] Cheong JE Sun L. Targeting the IDO1/TDO2–KYN–AhR Pathway for Cancer Immunotherapy – Challenges and Opportunities. Trends Pharmacol Sci (2018) 39:307–25. doi: 10.1016/j.tips.2017.11.007 29254698

[8] Munn DH Zhou M Attwood JT Bondarev I Conway SJ Marshall B. Prevention of Allogeneic Fetal Rejection by Tryptophan Catabolism. Science (1998) 281:1191–3. doi: 10.1126/science.281.5380.1191 9712583

[9] Munn DH Sharma MD Hou D Baban B Lee JR Antonia SJ. Expression of Indoleamine 2,3-Dioxygenase by Plasmacytoid Dendritic Cells in Tumor-Draining Lymph Nodes. J Clin Invest (2004) 114:280–90. doi: 10.1172/JCI21583 PMC449750 15254595

[10] Brandacher G Perathoner A Ladurner R Schneeberger S Obrist P Winkler C. Prognostic Value of Indoleamine 2,3-Dioxygenase Expression in Colorectal Cancer: Effect on Tumor-Infiltrating T Cells. Clin Cancer Res Off J Am Assoc Cancer Res (2006) 12:1144–51. doi: 10.1158/1078-0432.CCR-05-1966 16489067

[11] Opitz CA Litzenburger UM Sahm F Ott M Tritschler I Trump S. An Endogenous Tumour-Promoting Ligand of the Human Aryl Hydrocarbon Receptor. Nature (2011) 478:197–203. doi: 10.1038/nature10491 21976023

[12] Munn DH Mellor AL. Indoleamine 2,3 Dioxygenase and Metabolic Control of Immune Responses. Trends Immunol (2013) 34:137–43. doi: 10.1016/j.it.2012.10.001 PMC3594632 23103127

[13] Bessede A Gargaro M Pallotta MT Matino D Servillo G Brunacci C. Aryl Hydrocarbon Receptor Control of a Disease Tolerance Defence Pathway. Nature (2014) 511:184–90. doi: 10.1038/nature13323 PMC4098076 24930766

[14] Gutiérrez-Vázquez C Quintana FJ. Regulation of the Immune Response by the Aryl Hydrocarbon Receptor. Immunity (2018) 48:19–33. doi: 10.1016/j.immuni.2017.12.012 29343438 PMC5777317

[15] Prendergast GC Smith C Thomas S Mandik-Nayak L Laury-Kleintop L Metz R. Indoleamine 2,3-Dioxygenase Pathways of Pathogenic Inflammation and Immune Escape in Cancer. Cancer Immunol Immunother CII (2014) 63:721–35. doi: 10.1007/s00262-014-1549-4 PMC4384696 24711084

[16] Munn DH Mellor AL. IDO in the Tumor Microenvironment: Inflammation, Counter-Regulation, and Tolerance. Trends Immunol (2016) 37:193–207. doi: 10.1016/j.it.2016.01.002 26839260 PMC4916957

[17] Spranger S Koblish HK Horton B Scherle PA Newton R Gajewski TF. Mechanism of Tumor Rejection With Doublets of CTLA-4, PD-1/PD-L1, or IDO Blockade Involves Restored IL-2 Production and Proliferation of CD8(+) T Cells Directly Within the Tumor Microenvironment. J Immunother Cancer (2014) 2:3. doi: 10.1186/2051-1426-2-3 24829760 PMC4019906

[18] Badawy AA-B. Kynurenine Pathway of Tryptophan Metabolism: Regulatory and Functional Aspects. Int J Tryptophan Res IJTR (2017) 10:1178646917691938. doi: 10.1177/1178646917691938 28469468 PMC5398323

[19] Mondanelli G Mandarano M Belladonna ML Suvieri C Pelliccia C Bellezza G. Current Challenges for IDO2 as Target in Cancer Immunotherapy. Front Immunol (2021) 12:679953. doi: 10.3389/fimmu.2021.679953 33968089 PMC8097162

[20] Pallotta MT Orabona C Volpi C Vacca C Belladonna ML Bianchi R. Indoleamine 2,3-Dioxygenase is a Signaling Protein in Long-Term Tolerance by Dendritic Cells. Nat Immunol (2011) 12:870–8. doi: 10.1038/ni.2077 21804557

[21] Chen W. IDO: More Than an Enzyme. Nat Immunol (2011) 12:809–11. doi: 10.1038/ni.2088 21852775

[22] Konan KV Taylor MW. Importance of the Two Interferon-Stimulated Response Element (ISRE) Sequences in the Regulation of the Human Indoleamine 2,3-Dioxygenase Gene *. J Biol Chem (1996) 271:19140–5. doi: 10.1074/jbc.271.32.19140 8702590

[24] Litzenburger UM Opitz CA Sahm F Rauschenbach KJ Trump S Winter M. Constitutive IDO Expression in Human Cancer is Sustained by an Autocrine Signaling Loop Involving IL-6, STAT3 and the AHR. Oncotarget (2014) 5:1038–51. doi: 10.18632/oncotarget.1637 PMC4011581 24657910

[25] Ochs K Ott M Rauschenbach KJ Deumelandt K Sahm F Opitz CA. Tryptophan-2,3-Dioxygenase is Regulated by Prostaglandin E2 in Malignant Glioma *via* a Positive Signaling Loop Involving Prostaglandin E Receptor-4. J Neurochem (2016) 136:1142–54. doi: 10.1111/jnc.13503 26708701

[26] Liu X Shin N Koblish HK Yang G Wang Q Wang K. Selective Inhibition of IDO1 Effectively Regulates Mediators of Antitumor Immunity. Blood (2010) 115:3520–30. doi: 10.1182/blood-2009-09-246124 20197554

[27] Pilotte L Larrieu P Stroobant V Colau D Dolušić E Frédérick R. Reversal of Tumoral Immune Resistance by Inhibition of Tryptophan 2,3-Dioxygenase. Proc Natl Acad Sci (2012) 109:2497–502. doi: 10.1073/pnas.1113873109 PMC3289319 22308364

[28] Vigneron N van Baren N Van den Eynde BJ. Expression Profile of the Human IDO1 Protein, a Cancer Drug Target Involved in Tumoral Immune Resistance. OncoImmunology (2015) 4:e1003012. doi: 10.1080/2162402X.2014.1003012 26155395 PMC4485782

[29] Théate I van Baren N Pilotte L Moulin P Larrieu P Renauld J-C. Extensive Profiling of the Expression of the Indoleamine 2,3-Dioxygenase 1 Protein in Normal and Tumoral Human Tissues. Cancer Immunol Res (2015) 3:161–72. doi: 10.1158/2326-6066.CIR-14-0137 25271151

[30] Brochez L Chevolet I Kruse V. The Rationale of Indoleamine 2,3-Dioxygenase Inhibition for Cancer Therapy. Eur J Cancer Oxf Engl 1990 (2017) 76:167–82. doi: 10.1016/j.ejca.2017.01.011 28324751

[31] Clement CC D’Alessandro A Thangaswamy S Chalmers S Furtado R Spada S. 3-Hydroxy-L-Kynurenamine is an Immunomodulatory Biogenic Amine. Nat Commun (2021) 12:4447. doi: 10.1038/s41467-021-24785-3 34290243 PMC8295276

[32] Munn DH Shafizadeh E Attwood JT Bondarev I Pashine A Mellor AL. Inhibition of T Cell Proliferation by Macrophage Tryptophan Catabolism. J Exp Med (1999) 189:1363–72. doi: 10.1084/jem.189.9.1363 PMC2193062 10224276

[33] Lee GK Park HJ Macleod M Chandler P Munn DH Mellor AL. Tryptophan Deprivation Sensitizes Activated T Cells to Apoptosis Prior to Cell Division. Immunology (2002) 107:452–60. doi: 10.1046/j.1365-2567.2002.01526.x PMC1782830 12460190

[34] Fallarino F Grohmann U Vacca C Bianchi R Orabona C Spreca A. T Cell Apoptosis by Tryptophan Catabolism. Cell Death Differ (2002) 9:1069–77. doi: 10.1038/sj.cdd.4401073 12232795

[35] Munn DH Sharma MD Baban B Harding HP Zhang Y Ron D. GCN2 Kinase in T Cells Mediates Proliferative Arrest and Anergy Induction in Response to Indoleamine 2,3-Dioxygenase. Immunity (2005) 22:633–42. doi: 10.1016/j.immuni.2005.03.013 15894280

[36] Eleftheriadis T Pissas G Antoniadi G Liakopoulos V Stefanidis I. Indoleamine 2,3-Dioxygenase Depletes Tryptophan, Activates General Control non-Derepressible 2 Kinase and Down-Regulates Key Enzymes Involved in Fatty Acid Synthesis in Primary Human CD4+ T Cells. Immunology (2015) 146:292–300. doi: 10.1111/imm.12502 26147366 PMC4582970

[37] Fallarino F Grohmann U You S McGrath BC Cavener DR Vacca C. The Combined Effects of Tryptophan Starvation and Tryptophan Catabolites Down-Regulate T Cell Receptor Zeta-Chain and Induce a Regulatory Phenotype in Naive T Cells. J Immunol Baltim Md 1950 (2006) 176:6752–61. doi: 10.4049/jimmunol.176.11.6752 16709834

[38] Sonner JK Deumelandt K Ott M Thomé CM Rauschenbach KJ Schulz S. The Stress Kinase GCN2 Does Not Mediate Suppression of Antitumor T Cell Responses by Tryptophan Catabolism in Experimental Melanomas. OncoImmunology (2016) 5:e1240858. doi: 10.1080/2162402X.2016.1240858 28123877 PMC5214097

[39] Castilho BA Shanmugam R Silva RC Ramesh R Himme BM Sattlegger E. Keeping the Eif2 Alpha Kinase Gcn2 in Check. Biochim Biophys Acta (2014) 1843:1948–68. doi: 10.1016/j.bbamcr.2014.04.006 24732012

[40] DiNatale BC Murray IA Schroeder JC Flaveny CA Lahoti TS Laurenzana EM. Kynurenic Acid Is a Potent Endogenous Aryl Hydrocarbon Receptor Ligand That Synergistically Induces Interleukin-6 in the Presence of Inflammatory Signaling. Toxicol Sci (2010) 115:89–97. doi: 10.1093/toxsci/kfq024 20106948 PMC2855350

[41] Nguyen NT Kimura A Nakahama T Chinen I Masuda K Nohara K. Aryl Hydrocarbon Receptor Negatively Regulates Dendritic Cell Immunogenicity *via* a Kynurenine-Dependent Mechanism. Proc Natl Acad Sci (2010) 107:19961–6. doi: 10.1073/pnas.1014465107 PMC2993339 21041655

[42] Mezrich JD Fechner JH Zhang X Johnson BP Burlingham WJ Bradfield CA. An Interaction Between Kynurenine and the Aryl Hydrocarbon Receptor can Generate Regulatory T Cells. J Immunol Baltim Md 1950 (2010) 185:3190–8. doi: 10.4049/jimmunol.0903670 PMC2952546 20720200

[43] Rothhammer V Quintana FJ. The Aryl Hydrocarbon Receptor: An Environmental Sensor Integrating Immune Responses in Health and Disease. Nat Rev Immunol (2019) 19:184–97. doi: 10.1038/s41577-019-0125-8 30718831

[44] Sakurai S Shimizu T Ohto U. The Crystal Structure of the AhRR–ARNT Heterodimer Reveals the Structural Basis of the Repression of AhR-Mediated Transcription. J Biol Chem (2017) 292:17609–16. doi: 10.1074/jbc.M117.812974 PMC5663866 28904176

[45] Luecke-Johansson S Gralla M Rundqvist H Ho JC Johnson RS Gradin K. Poellinger L. A Molecular Mechanism To Switch the Aryl Hydrocarbon Receptor From a Transcription Factor to an E3 Ubiquitin Ligase. Mol Cell Biol (2017) 37:e00630-16. doi: 10.1128/MCB.00630-16 28416634 PMC5472827

[46] Vogel CFA Khan EM Leung PSC Gershwin ME Chang WLW Wu D. Cross-Talk Between Aryl Hydrocarbon Receptor and the Inflammatory Response: A Role for Nuclear Factor-κb. J Biol Chem (2014) 289:1866–75. doi: 10.1074/jbc.M113.505578 PMC3894361 24302727

[47] Kimura A Naka T Nakahama T Chinen I Masuda K Nohara K. Aryl Hydrocarbon Receptor in Combination With Stat1 Regulates LPS-Induced Inflammatory Responses. J Exp Med (2009) 206:2027–35. doi: 10.1084/jem.20090560 PMC2737163 19703987

[48] Seok S-H Ma Z-X Feltenberger JB Chen H Chen H Scarlett C. Trace Derivatives of Kynurenine Potently Activate the Aryl Hydrocarbon Receptor (AHR). J Biol Chem (2018) 293:1994–2005. doi: 10.1074/jbc.RA117.000631 29279331 PMC5808761

[49] Liu Y Liang X Dong W Fang Y Lv J Zhang T. Tumor-Repopulating Cells Induce PD-1 Expression in CD8+ T Cells by Transferring Kynurenine and AhR Activation. Cancer Cell (2018) 33:480–94.e7. doi: 10.1016/j.ccell.2018.02.005 29533786

[50] Greene LI Bruno TC Christenson JL D’Alessandro A Culp-Hill R Torkko K. Richer JK. A Role for Tryptophan-2,3-Dioxygenase in CD8 T-Cell Suppression and Evidence of Tryptophan Catabolism in Breast Cancer Patient Plasma. Mol Cancer Res MCR (2019) 17:131–9. doi: 10.1158/1541-7786.MCR-18-0362 PMC6318037 30143553

[51] Quintana FJ Murugaiyan G Farez MF Mitsdoerffer M Tukpah A-M Burns EJ. An Endogenous Aryl Hydrocarbon Receptor Ligand Acts on Dendritic Cells and T Cells to Suppress Experimental Autoimmune Encephalomyelitis. Proc Natl Acad Sci (2010) 107:20768–73. doi: 10.1073/pnas.1009201107 PMC2996442 21068375

[52] Wagage S John B Krock BL Hall AO Randall LM Karp CL. The Aryl Hydrocarbon Receptor Promotes IL-10 Production by NK Cells. J Immunol (2014) 192:1661–70. doi: 10.4049/jimmunol.1300497 PMC3955958 24403534

[53] Wang C Ye Z Kijlstra A Zhou Y Yang P. Activation of the Aryl Hydrocarbon Receptor Affects Activation and Function of Human Monocyte-Derived Dendritic Cells. Clin Exp Immunol (2014) 177:521–30. doi: 10.1111/cei.12352 PMC4226603 24749687

[54] Takenaka MC Gabriely G Rothhammer V Mascanfroni ID Wheeler MA Chao C-C. Control of Tumor-Associated Macrophages and T Cells in Glioblastoma *via* AHR and CD39. Nat Neurosci (2019) 22:729–40. doi: 10.1038/s41593-019-0370-y PMC8052632 30962630

[55] Apetoh L Quintana FJ Pot C Joller N Xiao S Kumar D. The Aryl Hydrocarbon Receptor Interacts With C-Maf to Promote the Differentiation of Type 1 Regulatory T Cells Induced by IL-27. Nat Immunol (2010) 11:854–61. doi: 10.1038/ni.1912 PMC2940320 20676095

[56] Tanaka A Sakaguchi S. Regulatory T Cells in Cancer Immunotherapy. Cell Res (2017) 27:109–18. doi: 10.1038/cr.2016.151 PMC5223231 27995907

[57] Kumar V Patel S Tcyganov E Gabrilovich DI. The Nature of Myeloid-Derived Suppressor Cells in the Tumor Microenvironment. Trends Immunol (2016) 37:208–20. doi: 10.1016/j.it.2016.01.004 PMC4775398 26858199

[58] Yang L Zhang Y. Tumor-Associated Macrophages: From Basic Research to Clinical Application. J Hematol OncolJ Hematol Oncol (2017) 10:58. doi: 10.1186/s13045-017-0430-2 28241846 PMC5329931

[59] Manlapat AK Kahler DJ Chandler PR Munn DH Mellor AL. Cell-Autonomous Control of Interferon Type I Expression by Indoleamine 2,3-Dioxygenase in Regulatory CD19+ Dendritic Cells. Eur J Immunol (2007) 37:1064–71. doi: 10.1002/eji.200636690 17343295

[60] Yamada T Horimoto H Kameyama T Hayakawa S Yamato H Dazai M. Constitutive Aryl Hydrocarbon Receptor Signaling Constrains Type I Interferon–Mediated Antiviral Innate Defense. Nat Immunol (2016) 17:687–94. doi: 10.1038/ni.3422 27089381

[61] Quintana FJ Basso AS Iglesias AH Korn T Farez MF Bettelli E. Control of T Reg and T H 17 Cell Differentiation by the Aryl Hydrocarbon Receptor. Nature (2008) 453:65–71. doi: 10.1038/nature06880 18362915

[62] Yeste A Nadeau M Burns EJ Weiner HL Quintana FJ. Nanoparticle-Mediated Codelivery of Myelin Antigen and a Tolerogenic Small Molecule Suppresses Experimental Autoimmune Encephalomyelitis. Proc Natl Acad Sci (2012) 109:11270–5. doi: 10.1073/pnas.1120611109 PMC3396465 22745170

[63] Kwiatkowska I Hermanowicz JM Przybyszewska-Podstawka A Pawlak D. Not Only Immune Escape—The Confusing Role of the TRP Metabolic Pathway in Carcinogenesis. Cancers (2021) 13:2667. doi: 10.3390/cancers13112667 34071442 PMC8198784

[64] Shin JH Zhang L Murillo-Sauca O Kim J Kohrt HEK Bui JD. Modulation of Natural Killer Cell Antitumor Activity by the Aryl Hydrocarbon Receptor. Proc Natl Acad Sci (2013) 110:12391–6. doi: 10.1073/pnas.1302856110 PMC3725066 23836658

[65] Li J Doty A Glover SC. Aryl Hydrocarbon Receptor Activation and IL-2 Have Synergistic Effects That Enhance Human Natural Killer Cell Cytotoxicity. J Immunol (2016) 196:129.11–1. doi: 10.14800/ics.1404

[66] Goudot C Coillard A Villani A-C Gueguen P Cros A Sarkizova S. Aryl Hydrocarbon Receptor Controls Monocyte Differentiation Into Dendritic Cells Versus Macrophages. Immunity (2017) 47:582–96.e6. doi: 10.1016/j.immuni.2017.08.016 28930664

[67] Chevolet I Speeckaert R Schreuer M Neyns B Krysko O Bachert C. Characterization of the *In Vivo* Immune Network of IDO, Tryptophan Metabolism, PD-L1, and CTLA-4 in Circulating Immune Cells in Melanoma. OncoImmunology (2015) 4:e982382. doi: 10.4161/2162402X.2014.982382 25949897 PMC4404886

[68] Spranger S Spaapen RM Zha Y Williams J Meng Y Ha TT. Up-Regulation of PD-L1, IDO, and Tregs in the Melanoma Tumor Microenvironment Is Driven by CD8+ T Cells. Sci Transl Med (2013) 5:200ra116–200ra116. doi: 10.1126/scitranslmed.3006504 PMC4136707 23986400

[69] Fallarino F Grohmann U Hwang KW Orabona C Vacca C Bianchi R. Modulation of Tryptophan Catabolism by Regulatory T Cells. Nat Immunol (2003) 4:1206–12. doi: 10.1038/ni1003 14578884

[70] Rodrigues CP Ferreira ACF Pinho MP de Moraes CJ Bergami-Santos PC Barbuto JAM. Tolerogenic IDO(+) Dendritic Cells Are Induced by PD-1-Expressing Mast Cells. Front Immunol (2016) 7:9. doi: 10.3389/fimmu.2016.00009 26834749 PMC4724729

[71] Sharma MD Shinde R McGaha TL Huang L Holmgaard RB Wolchok JD. The PTEN Pathway in Tregs is a Critical Driver of the Suppressive Tumor Microenvironment. Sci Adv (2015) 1:e1500845. doi: 10.1126/sciadv.1500845 26601142 PMC4640592

[72] Amobi-McCloud A Muthuswamy R Battaglia S Yu H Liu T Wang J. IDO1 Expression in Ovarian Cancer Induces PD-1 in T Cells *via* Aryl Hydrocarbon Receptor Activation. Front Immunol (2021) 12:678999:678999. doi: 10.3389/fimmu.2021.678999 34025677 PMC8136272

[73] Holmgaard RB Zamarin D Munn DH Wolchok JD Allison JP. Indoleamine 2,3-Dioxygenase is a Critical Resistance Mechanism in Antitumor T Cell Immunotherapy Targeting CTLA-4. J Exp Med (2013) 210:1389–402. doi: 10.1084/jem.20130066 PMC3698523 23752227

[74] Wainwright DA Chang AL Dey M Balyasnikova IV Kim CK Tobias A. Durable Therapeutic Efficacy Utilizing Combinatorial Blockade Against IDO, CTLA-4, and PD-L1 in Mice With Brain Tumors. Clin Cancer Res Off J Am Assoc Cancer Res (2014) 20:5290–301. doi: 10.1158/1078-0432.CCR-14-0514 PMC4182350 24691018

[75] Labadie BW Bao R Luke JJ. Reimagining IDO Pathway Inhibition in Cancer Immunotherapy *via* Downstream Focus on the Tryptophan–Kynurenine–Aryl Hydrocarbon Axis. Clin Cancer Res (2019) 25:1462–71. doi: 10.1158/1078-0432.CCR-18-2882 PMC6397695 30377198

[76] Smith C Chang MY Parker KH Beury DW DuHadaway JB Flick HE. IDO is a Nodal Pathogenic Driver of Lung Cancer and Metastasis Development. Cancer Discovery (2012) 2:722–35. doi: 10.1158/2159-8290.CD-12-0014 PMC3677576 22822050

[77] Uyttenhove C Pilotte L Théate I Stroobant V Colau D Parmentier N. Evidence for a Tumoral Immune Resistance Mechanism Based on Tryptophan Degradation by Indoleamine 2,3-Dioxygenase. Nat Med (2003) 9:1269–74. doi: 10.1038/nm934 14502282

[78] Godin-Ethier J Hanafi L-A Piccirillo CA Lapointe R. Indoleamine 2,3-Dioxygenase Expression in Human Cancers: Clinical and Immunologic Perspectives. Clin Cancer Res (2011) 17:6985–91. doi: 10.1158/1078-0432.CCR-11-1331 22068654

[79] Yue EW Sparks R Polam P Modi D Douty B Wayland B. INCB24360 (Epacadostat), a Highly Potent and Selective Indoleamine-2,3-Dioxygenase 1 (IDO1) Inhibitor for Immuno-Oncology. ACS Med Chem Lett (2017) 8:486–91. doi: 10.1021/acsmedchemlett.6b00391 PMC5430407 28523098

[80] Jochems C Fantini M Fernando RI Kwilas AR Donahue RN Lepone LM. The IDO1 Selective Inhibitor Epacadostat Enhances Dendritic Cell Immunogenicity and Lytic Ability of Tumor Antigen-Specific T Cells. Oncotarget (2016) 7:37762–72. doi: 10.18632/oncotarget.9326 PMC5122347 27192116

[81] Koblish HK Hansbury MJ Bowman KJ Yang G Neilan CL Haley PJ. Hydroxyamidine Inhibitors of Indoleamine-2,3-Dioxygenase Potently Suppress Systemic Tryptophan Catabolism and the Growth of IDO-Expressing Tumors. Mol Cancer Ther (2010) 9:489–98. doi: 10.1158/1535-7163.MCT-09-0628 20124451

[82] Reardon DA Gokhale PC Klein SR Jones KL Kirschmeier PT Speranza M. Abstract 572: Inhibition of IDO1 With Epacadostat Enhances Anti-Tumor Efficacy of PD-1 Blockade in a Syngeneic Glioblastoma (GBM) Model. Cancer Res (2017) 77:572–2. doi: 10.1158/1538-7445.AM2017-572

[83] Beatty GL O’Dwyer PJ Clark J Shi JG Bowman KJ Scherle PA. First-In-Human Phase I Study of the Oral Inhibitor of Indoleamine 2,3-Dioxygenase-1 Epacadostat (INCB024360) in Patients With Advanced Solid Malignancies. Clin Cancer Res Off J Am Assoc Cancer Res (2017) 23:3269–76. doi: 10.1158/1078-0432.CCR-16-2272 PMC5496788 28053021

[84] Komrokji RS Wei S Mailloux AW Zhang L Padron E Lancet JE. A Phase II Study to Determine the Safety and Efficacy of the Oral Inhibitor of Indoleamine 2,3-Dioxygenase (IDO) Enzyme INCB024360 in Patients With Myelodysplastic Syndromes. Blood (2014) 124:4653–3. doi: 10.1182/blood.V124.21.4653.4653 30713125

[85] Kristeleit R Davidenko I Shirinkin V El-Khouly F Bondarenko I Goodheart MJ. A Randomised, Open-Label, Phase 2 Study of the IDO1 Inhibitor Epacadostat (INCB024360) Versus Tamoxifen as Therapy for Biochemically Recurrent (CA-125 Relapse)–Only Epithelial Ovarian Cancer, Primary Peritoneal Carcinoma, or Fallopian Tube Cancer. Gynecol Oncol (2017) 146:484–90. doi: 10.1016/j.ygyno.2017.07.005 28698009

[86] Long GV Dummer R Hamid O Gajewski TF Caglevic C Dalle S. Epacadostat Plus Pembrolizumab Versus Placebo Plus Pembrolizumab in Patients With Unresectable or Metastatic Melanoma (ECHO-301/KEYNOTE-252): A Phase 3, Randomised, Double-Blind Study. Lancet Oncol (2019) 20:1083–97. doi: 10.1016/S1470-2045(19)30274-8 31221619

[87] Mautino MR Jaipuri FA Waldo J Kumar S Adams J Allen CV. Abstract 491: NLG919, a Novel Indoleamine-2,3-Dioxygenase (IDO)-Pathway Inhibitor Drug Candidate for Cancer Therapy. Cancer Res (2013) 73:491–1. doi: 10.1158/1538-7445.AM2013-491

[88] Mautino MR Link CJ Vahanian NN Adams JT Allen CV Sharma MD. Abstract 5023: Synergistic Antitumor Effects of Combinatorial Immune Checkpoint Inhibition With Anti-PD-1/PD-L Antibodies and the IDO Pathway Inhibitors NLG-919 and Indoximod in the Context of Active Immunotherapy. Cancer Res (2014) 74:5023–3. doi: 10.1158/1538-7445.AM2014-5023

[89] Spahn J Peng J Lorenzana E Kan D Hunsaker T Segal E. Improved Anti-Tumor Immunity and Efficacy Upon Combination of the IDO1 Inhibitor GDC-0919 With Anti-PD-L1 Blockade Versus Anti-PD-L1 Alone in Preclinical Tumor Models. J Immunother Cancer (2015) 3:P303. doi: 10.1186/2051-1426-3-S2-P303

[90] Jung KH LoRusso P Burris H Gordon M Bang Y-J Hellmann MD. Phase I Study of the Indoleamine 2,3-Dioxygenase 1 (IDO1) Inhibitor Navoximod (GDC-0919) Administered With PD-L1 Inhibitor (Atezolizumab) in Advanced Solid Tumors. Clin Cancer Res (2019) 25:3220–8. doi: 10.1158/1078-0432.CCR-18-2740 PMC7980952 30770348

[91] Hunt JT Balog A Huang C Lin T-A Lin T-A Maley D. Abstract 4964: Structure, *In Vitro* Biology and *In Vivo* Pharmacodynamic Characterization of a Novel Clinical IDO1 Inhibitor. Cancer Res (2017) 77:4964–4. doi: 10.1158/1538-7445.AM2017-4964

[92] Siu LL Gelmon K Chu Q Pachynski R Alese O Basciano P. Abstract CT116: BMS-986205, an Optimized Indoleamine 2,3-Dioxygenase 1 (IDO1) Inhibitor, is Well Tolerated With Potent Pharmacodynamic (PD) Activity, Alone and in Combination With Nivolumab (Nivo) in Advanced Cancers in a Phase 1/2a Trial. Cancer Res (2017) 77:CT116–6. doi: 10.1158/1538-7445.AM2017-CT116

[93] Reardon DA Desjardins A Rixe O Cloughesy T Alekar S Williams JH. A Phase 1 Study of PF-06840003, an Oral Indoleamine 2,3-Dioxygenase 1 (IDO1) Inhibitor in Patients With Recurrent Malignant Glioma. Invest New Drugs (2020) 38:1784–95. doi: 10.1007/s10637-020-00950-1 32436060

[94] Tumang J Gomes B Wythes M Crosignani S Bingham P Bottemanne P. Abstract 4863: PF-06840003: A Highly Selective IDO-1 Inhibitor That Shows Good *In Vivo* Efficacy in Combination With Immune Checkpoint Inhibitors. Cancer Res (2016) 76:4863–3. doi: 10.1158/1538-7445.AM2016-4863

[95] Hou D-Y Muller AJ Sharma MD DuHadaway J Banerjee T Johnson M. Inhibition of Indoleamine 2,3-Dioxygenase in Dendritic Cells by Stereoisomers of 1-Methyl-Tryptophan Correlates With Antitumor Responses. Cancer Res (2007) 67:792–801. doi: 10.1158/0008-5472.CAN-06-2925 17234791

[96] Metz R Rust S DuHadaway JB Mautino MR Munn DH Vahanian NN. IDO Inhibits a Tryptophan Sufficiency Signal That Stimulates mTOR: A Novel IDO Effector Pathway Targeted by D-1-Methyl-Tryptophan. OncoImmunology (2012) 1:1460–8. doi: 10.4161/onci.21716 PMC3525601 23264892

[97] Brincks EL Adams J Wang L Turner B Marcinowicz A Ke J. Indoximod Opposes the Immunosuppressive Effects Mediated by IDO and TDO *via* Modulation of AhR Function and Activation of Mtorc1. Oncotarget (2020) 11:2438–61. doi: 10.18632/oncotarget.27646 PMC7321702 32637034

[98] Fox E Oliver T Rowe M Thomas S Zakharia Y Gilman PB. Indoximod: An Immunometabolic Adjuvant That Empowers T Cell Activity in Cancer. Front Oncol (2018) 8:370. doi: 10.3389/fonc.2018.00370 30254983 PMC6141803

[99] Colwell J. Indoximod Combo Triggers Responses in Melanoma. Cancer Discov (2017) 7:542–3. doi: 10.1158/2159-8290.CD-NB2017-056 28381404

[100] Zakharia Y McWilliams RR Rixe O Drabick J Shaheen MF Grossmann KF. Phase II Trial of the IDO Pathway Inhibitor Indoximod Plus Pembrolizumab for the Treatment of Patients With Advanced Melanoma. J Immunother Cancer (2021) 9:e002057. doi: 10.1136/jitc-2020-002057 34117113 PMC8202104

[101] Kumar S Jaipuri FA Waldo JP Potturi H Marcinowicz A Adams J. Discovery of Indoximod Prodrugs and Characterization of Clinical Candidate NLG802. Eur J Med Chem (2020) 198:112373. doi: 10.1016/j.ejmech.2020.112373 32422549

[102] Kotecki N Vuagnat P O’Neil BH Jalal S Rottey S Prenen H. A Phase I Study of an IDO-1 Inhibitor (LY3381916) as Monotherapy and in Combination With an Anti-PD-L1 Antibody (LY3300054) in Patients With Advanced Cancer. J Immunother Hagerstown Md 1997 (2021) 144(7):264–75. doi: 10.1097/CJI.0000000000000368 33928928

[103] Kjeldsen JW Lorentzen CL Martinenaite E Ellebaek E Donia M Holmstroem RB. A Phase 1/2 Trial of an Immune-Modulatory Vaccine Against IDO/PD-L1 in Combination With Nivolumab in Metastatic Melanoma. Nat Med (2021) 27:2212–23. doi: 10.1038/s41591-021-01544-x PMC8904254 34887574

[104] Sherr D Kenison-Whte J Wang Z. Abstract LB-128: The Aryl Hydrocarbon Receptor as Driver of Cancer Immunity. Cancer Res (2018) 78:LB-LB-128. doi: 10.1158/1538-7445.AM2018-LB-128

[105] Joseph J Gonzalez-Lopez M Galang C Garcia C Lemar H Lu J. Abstract 4719: Small-Molecule Antagonists of the Aryl Hydrocarbon Receptor (AhR) Promote Activation of Human PBMCs *In Vitro* and Demonstrate Significant Impact on Tumor Growth and Immune Modulation *In Vivo* . Cancer Res (2018) 78:4719–9. doi: 10.1158/1538-7445.AM2018-4719

[106] Zhang M Stone E Triplett TA Triplett K Lamb C Karamitros CS. Abstract 5570: A Novel Approach to Targeting the IDO/TDO Pathway Through Degradation of the Immunosuppressive Metabolite Kynurenine. Cancer Res (2017) 77:5570–0. doi: 10.1158/1538-7445.AM2017-5570

[107] Triplett TA Triplett K Stone E Zhang M Manfredi M Lamb C. Abstract 5571: Immune-Checkpoint Inhibition *via* Enzyme-Mediated Degradation of Kynurenine. Cancer Res (2017) 77:5571–1. doi: 10.1158/1538-7445.AM2017-5571

[108] Stone E Marshall N Donkor M Triplett K Blazek J Triplett T. Abstract LB-226: Depletion of Kynurenine Using an Engineered Therapeutic Enzyme Potently Inhibits Cancer Immune Checkpoints Both as a Monotherapy and in Combination With Anti-PD-1. Cancer Res (2015) 75:LB-LB-226. doi: 10.1158/1538-7445.AM2015-LB-226

[109] Triplett TA Garrison KC Marshall N Donkor M Blazeck J Lamb C. Reversal of Indoleamine 2,3-Dioxygenase–Mediated Cancer Immune Suppression by Systemic Kynurenine Depletion With a Therapeutic Enzyme. Nat Biotechnol (2018) 36:758–64. doi: 10.1038/nbt.4180 PMC6078800 30010674

[110] Murray IA Patterson AD Perdew GH. Aryl Hydrocarbon Receptor Ligands In Cancer: Friend and Foe. Nat Rev Cancer (2014) 14:801–14. doi: 10.1038/nrc3846 PMC4401080 25568920

[111] Platten M Wick W Van den Eynde BJ. Tryptophan Catabolism in Cancer: Beyond IDO and Tryptophan Depletion. Cancer Res (2012) 72:5435–40. doi: 10.1158/0008-5472.CAN-12-0569 23090118

[112] Gargaro M Manni G Scalisi G Puccetti P Fallarino F. Tryptophan Metabolites at the Crossroad of Immune-Cell Interaction *via* the Aryl Hydrocarbon Receptor: Implications for Tumor Immunotherapy. Int J Mol Sci (2021) 22:4644. doi: 10.3390/ijms22094644 33924971 PMC8125364

[113] Soshilov AA Denison MS. Ligand Promiscuity of Aryl Hydrocarbon Receptor Agonists and Antagonists Revealed by Site-Directed Mutagenesis. Mol Cell Biol (2014) 34:1707–19. doi: 10.1128/MCB.01183-13 PMC3993610 24591650

[114] Nguyen LP Bradfield CA. The Search for Endogenous Activators of the Aryl Hydrocarbon Receptor. Chem Res Toxicol (2008) 21:102–16. doi: 10.1021/tx7001965 PMC2572005 18076143

[115] Tigges J Haarmann-Stemmann T Vogel CFA Grindel A Hübenthal U Brenden H. The New Aryl Hydrocarbon Receptor Antagonist E/Z-2-Benzylindene-5,6-Dimethoxy-3,3-Dimethylindan-1-One Protects Against UVB-Induced Signal Transduction. J Invest Dermatol (2014) 134:556–9. doi: 10.1038/jid.2013.362 PMC4005890 23995519

[116] Parks AJ Pollastri MP Hahn ME Stanford EA Novikov O Franks DG. In Silico Identification of an Aryl Hydrocarbon Receptor Antagonist With Biological Activity *In Vitro* and *In Vivo* . Mol Pharmacol (2014) 86:593–608. doi: 10.1124/mol.114.093369 25159092 PMC4201140

[117] Pinto S Steeneck C Albers M Anderhub S Birkel M Buselic-Wölfel L. Abstract 1210: Targeting the IDO1-Kynurenine-AhR Pathway for Cancer Immunotherapy. Cancer Res (2019) 79:1210–0. doi: 10.1158/1538-7445.AM2019-1210

[118] Gutcher I Kober C Roese L Roewe J Schmees N Prinz F. Abstract 1288: Blocking Tumor-Associated Immune Suppression With BAY-218, a Novel, Selective Aryl Hydrocarbon Receptor (AhR) Inhibitor. Cancer Res (2019) 79:1288–8. doi: 10.1158/1538-7445.AM2019-1288

[119] Schmees N Gutcher I Roehn U Irlbacher H Stoeckigt D Bader B. Abstract 4454: Identification of BAY-218, a Potent and Selective Small-Molecule AhR Inhibitor, as a New Modality to Counteract Tumor Immunosuppression. Cancer Res (2019) 79:4454–4. doi: 10.1158/1538-7445.AM2019-4454

[120] Hall JM Barhoover MA Kazmin D McDonnell DP Greenlee WF Thomas RS. Activation of the Aryl-Hydrocarbon Receptor Inhibits Invasive and Metastatic Features of Human Breast Cancer Cells and Promotes Breast Cancer Cell Differentiation. Mol Endocrinol (2010) 24:359–69. doi: 10.1210/me.2009-0346 PMC2817602 20032195

[121] Prud’homme GJ Glinka Y Toulina A Ace O Subramaniam V Jothy S. Breast Cancer Stem-Like Cells Are Inhibited by a Non-Toxic Aryl Hydrocarbon Receptor Agonist. PloS One (2010) 5:e13831. doi: 10.1371/journal.pone.0013831 21072210 PMC2972222

[122] Wang T Wyrick KL Meadows GG Wills TB Vorderstrasse BA. Activation of the Aryl Hydrocarbon Receptor by TCDD Inhibits Mammary Tumor Metastasis in a Syngeneic Mouse Model of Breast Cancer. Toxicol Sci (2011) 124:291–8. doi: 10.1093/toxsci/kfr247 PMC3216416 21948867

[123] Moyer BJ Rojas IY Murray IA Lee S Hazlett HF Perdew GH. Indoleamine 2,3-Dioxygenase 1 (IDO1) Inhibitors Activate the Aryl Hydrocarbon Receptor. Toxicol Appl Pharmacol (2017) 323:74–80. doi: 10.1016/j.taap.2017.03.012 28336214 PMC5495139

[124] Zhai L Spranger S Binder DC Gritsina G Lauing KL Giles FJ. Molecular Pathways: Targeting IDO1 and Other Tryptophan Dioxygenases for Cancer Immunotherapy. Clin Cancer Res (2015) 21:5427–33. doi: 10.1158/1078-0432.CCR-15-0420 PMC4681601 26519060

[125] Zhai L Ladomersky E Lenzen A Nguyen B Patel R Lauing KL. IDO1 in Cancer: A Gemini of Immune Checkpoints. Cell Mol Immunol (2018) 15:447–57. doi: 10.1038/cmi.2017.143 PMC6068130 29375124

[126] Schollbach J Löb S. Challenges in Targeting the Tryptophan Metabolism Incancer Immunotherapy. Aging (2021) 13:19952–3. doi: 10.18632/aging.203492 PMC8436902 34446610

[127] Abdel-Magid AF. Targeting the Inhibition of Tryptophan 2,3-Dioxygenase (TDO-2) for Cancer Treatment. ACS Med Chem Lett (2017) 8:11–3. doi: 10.1021/acsmedchemlett.6b00458 PMC5238467 28105265

[128] Larkin PB Sathyasaikumar KV Notarangelo FM Funakoshi H Nakamura T Schwarcz R. Tryptophan 2,3-Dioxygenase and Indoleamine 2,3-Dioxygenase 1 Make Separate, Tissue-Specific Contributions to Basal and Inflammation-Induced Kynurenine Pathway Metabolism in Mice. Biochim Biophys Acta (2016) 1860:2345–54. doi: 10.1016/j.bbagen.2016.07.002 PMC5808460 27392942

[129] Gyulveszi G Fischer C Mirolo M Stern M Green L Ceppi M. Abstract LB-085: RG70099: A Novel, Highly Potent Dual IDO1/TDO Inhibitor to Reverse Metabolic Suppression of Immune Cells in the Tumor Micro-Environment. Cancer Res (2016) 76:LB-LB-085. doi: 10.1158/1538-7445.AM2016-LB-085

[130] Gullapalli S Roychowdhury A Khaladkar T Sawargave S Janrao R Kalhapure V. Abstract 1701: EPL-1410, a Novel Fused Heterocycle Based Orally Active Dual Inhibitor of IDO1/TDO2, as a Potential Immune-Oncology Therapeutic. Cancer Res (2018) 78:1701–1. doi: 10.1158/1538-7445.AM2018-1701

[131] Kim C Kim JH Kim JS Chon HJ Kim J-H. A Novel Dual Inhibitor of IDO and TDO, CMG017, Potently Suppresses the Kynurenine Pathway and Overcomes Resistance to Immune Checkpoint Inhibitors. J Clin Oncol (2019) 37:e14228–8. doi: 10.1200/JCO.2019.37.15_suppl.e14228

[132] York A. Microbiome: Gut Microbiota Sways Response to Cancer Immunotherapy. Nat Rev Microbiol (2018) 16:121. doi: 10.1038/nrmicro.2018.12 29355853

[133] Routy B Le Chatelier E Derosa L Duong CPM Alou MT Daillère R. Gut Microbiome Influences Efficacy of PD-1-Based Immunotherapy Against Epithelial Tumors. Science (2018) 359:91–7. doi: 10.1126/science.aan3706 29097494

[134] Gao J Xu K Liu H Liu G Bai M Peng C. Impact of the Gut Microbiota on Intestinal Immunity Mediated by Tryptophan Metabolism. Front Cell Infect Microbiol (2018) 8:13. doi: 10.3389/fcimb.2018.00013 29468141 PMC5808205

[135] Xie L Hu K Duo Y Shimokawa T Kumata K Zhang Y. Off-Tumor IDO1 Target Engagements Determine the Cancer-Immune Set Point and Predict the Immunotherapeutic Efficacy. J Immunother Cancer (2021) 9:e002616. doi: 10.1136/jitc-2021-002616 34148865 PMC8237741

[136] Li H Bullock K Gurjao C Braun D Shukla SA Bossé D. Metabolomic Adaptations and Correlates of Survival to Immune Checkpoint Blockade. Nat Commun (2019) 10:4346. doi: 10.1038/s41467-019-12361-9 31554815 PMC6761178

[137] Botticelli A Mezi S Pomati G Cerbelli B Cerbelli E Roberto M. Tryptophan Catabolism as Immune Mechanism of Primary Resistance to Anti-PD-1. Front Immunol (2020) 11:1243. doi: 10.3389/fimmu.2020.01243 32733441 PMC7358280

